# Inferring species richness using multispecies occupancy modeling: Estimation performance and interpretation

**DOI:** 10.1002/ece3.4821

**Published:** 2019-02-05

**Authors:** Gurutzeta Guillera‐Arroita, Marc Kéry, José J. Lahoz‐Monfort

**Affiliations:** ^1^ School of BioSciences University of Melbourne Parkville Victoria Australia; ^2^ Swiss Ornithological Institute Sempach Switzerland

**Keywords:** data augmentation, detectability, imperfect detection, richness, species occupancy, Switzerland

## Abstract

Multispecies occupancy models can estimate species richness from spatially replicated multispecies detection/non‐detection survey data, while accounting for imperfect detection. A model extension using data augmentation allows inferring the *total* number of species in the community, including those completely missed by sampling (i.e.*,* not detected in any survey, at any site). Here we investigate the robustness of these estimates. We review key model assumptions and test performance via simulations, under a range of scenarios of species characteristics and sampling regimes, exploring sensitivity to the Bayesian priors used for model fitting. We run tests when assumptions are perfectly met and when violated. We apply the model to a real dataset and contrast estimates obtained with and without predictors, and for different subsets of data. We find that, even with model assumptions perfectly met, estimation of the total number of species can be poor in scenarios where many species are missed (>15%–20%) and that commonly used priors can accentuate overestimation. Our tests show that estimation can often be robust to violations of assumptions about the statistical distributions describing variation of occupancy and detectability among species, but lower‐tail deviations can result in large biases. We obtain substantially different estimates from alternative analyses of our real dataset, with results suggesting that missing relevant predictors in the model can result in richness underestimation. In summary, estimates of total richness are sensitive to model structure and often uncertain. Appropriate selection of priors, testing of assumptions, and model refinement are all important to enhance estimator performance. Yet, these do not guarantee accurate estimation, particularly when many species remain undetected. While statistical models can provide useful insights, expectations about accuracy in this challenging prediction task should be realistic. Where knowledge about species numbers is considered truly critical for management or policy, survey effort should ideally be such that the chances of missing species altogether are low.

## 1 INTRODUCTION

Species richness, that is, number of species at a location, is a fundamental biodiversity measure that underlies many ecological questions and conservation decisions (Gotelli & Colwell, 2010). Countless studies seek to elucidate patterns and drivers of species richness (e.g., Fraser et al., 2015; Woolley et al., 2016), and identifying areas of high diversity is relevant for conservation prioritization (Fleishman, Noss, & Noon, 2006). Statistical models of richness can assist in these tasks (Ferrier & Guisan, 2006). By relating richness (or its components) to environmental conditions, such models allow formal inference about richness–environment relationships and can be used for prediction to areas that have not been surveyed.

Traditionally, studies of richness patterns have often been based on metrics obtained from raw “presence–absence” data. One modeling approach has been to relate directly spatially replicated species counts to the values of environmental predictors at those sites, following a “stack‐first‐predict‐later” strategy (Ferrier & Guisan, 2006). Another approach describes the raw “presence/absence” of individual species as a function of predictors and obtains richness estimates by stacking the resulting individual species models (Calabrese, Certain, Kraan, & Dormann, 2014; Ferrier & Guisan, 2006). In this “predict‐first‐stack‐later” strategy, richness is thus an emergent property. Both approaches assume that species are perfectly detected at sites where they are present and, consequently, only consider the species recorded during the sampling. However, imperfect detection is an almost ubiquitous issue in ecological data (Kellner & Swihart, 2014). It is easy for species to remain undetected during surveys (Kéry & Plattner, 2007), including for sessile taxa such as plants (Chen, Kéry, Plattner, Ma, & Gardner, 2013). Species may not be detected either because they are not present at the specific locations sampled (though they occur nearby), or because observers fail to record them where present. Species detectability can vary in space, time, and among taxa (Guillera‐Arroita, 2017; Iknayan, Tingley, Furnas, & Beissinger, 2014); it depends not only on characteristics of the species and their environment, but also on survey methods, effort, and very much on the observer. Studies and comparisons of species richness that disregard detectability risk that real patterns are masked or spurious patterns falsely identified (Gotelli & Colwell, 2001; Iknayan et al., 2014; Jarzyna & Jetz, 2016; Tingley & Beissinger, 2013).

A large number of methods have been developed to quantify species richness and account for species missed in sampling. Rarefaction methods avoid some of the pitfalls in richness comparisons between sites by addressing the effects of abundance and sampling effort on species counts (Gotelli & Colwell, 2001), yet they still make unrealistic assumptions about detectability (that is constant across samples, observers, and species; Gotelli & Ellison, 2012, p. 469). Rarefaction is a method of interpolation and hence does not provide estimates of “asymptotic” richness (the actual number of species at the site). Several estimation methods have been proposed for this task, including parametric and non‐parametric (capture–recapture‐based) approaches (for an overview see Gotelli & Colwell, 2010, section 4.2.8). These methods work by considering the frequencies (incidence or abundance) at which detected species have been observed. Based on this information, they infer the likely number of species present but unrecorded. Parametric approaches do so by fitting a parametric distribution to the whole set of observed frequencies; non‐parametric methods focus on species with few detections and include variants that account for heterogeneity in detectability among species (Boulinier, Nichols, Sauer, Hines, & Pollock, 1998; Burnham & Overton, 1979). All these estimation methods operate at the level of a “site” (meaning they yield a single richness estimate for the chosen area of inference). Where the aim is to build a model of spatial variation in species richness, estimates obtained at different sites can be treated as the response variable, and related to environmental predictors in a second stage of analysis (e.g., Brehm, Colwell, & Kluge, 2007). However, taking estimates as true values is problematic as their uncertainty is typically disregarded. Kéry and Royle (2016, pp. 679–682) propose to follow meta‐analytical principles to propagate uncertainty in such two‐step modeling process, but this requires assumptions about the error distributions.

Arguably, a more desirable approach to modeling spatial variation in richness is one that allows both accounting for species detectability flexibly and describing the pattern directly as a function of site characteristics thus avoiding separate stages in the analyses. With this philosophy, Dorazio and Royle (2005) proposed to approach richness modeling (or more generally, community modeling) by “stacking” single‐species occupancy–detection models (for a related, independent development of similar models see Gelfand et al., 2005). These building units are models of species occurrence that account for imperfect detection (Guillera‐Arroita, 2017; MacKenzie et al., 2002). The model combines the individual species occurrence predictions to derive richness (or other community metrics), resulting thus in an extension of the “predict‐first‐stack‐later” strategy mentioned above. This structure allows accommodating different species responses to environmental covariates, also in the detectability component, which cannot be achieved if observations are aggregated to model observed richness directly as a function of predictors. Furthermore, the approach allows an extension to infer the total number of species in the community, including those completely missed during the sampling (i.e., species not detected in any of the surveys, at any of the sites).

The multispecies occupancy–detection modeling framework and, in particular, its extension to infer the total number of species in the community (hereafter *N*) are the focus of this paper. This modeling framework is gaining uptake (see Supporting Information Appendix S1 for a literature review on its usage), including further extensions (e.g., Sutherland, Brambilla, Pedrini, & Tenan, 2016, for inference about geographic variation in species richness). However, there has been comparatively little effort put into exploring its properties, and in particular into assessing how well *N* can be inferred under different scenarios (but see Broms, Hooten, & Fitzpatrick, 2015; and Yamaura, Kéry, Kéry, & Royle, 2016, for related work on abundance community models). Our paper aims to progress in this direction.

**Figure 1:**
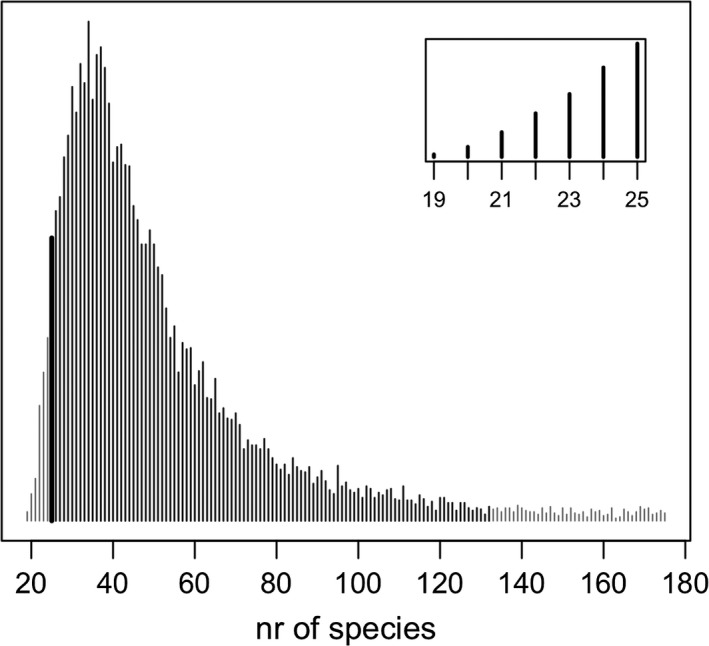
Reanalysis of the bog ant data in Dorazio et al. (2011), relaxing the imposed upper limit for estimated number of species (from 25 to 175). Based on the data alone, the Bayesian posterior suggests estimated richness substantially higher than expected by experts (median = 43, equal‐tailed 95% credible interval = [24,133]; compared to 25 species, highlighted by a thick line above). The surveys detected 19 species. Original estimation (with prior constrained to 25) shown in the inset. Details about the data are provided in Dorazio et al. (2011), together with the computer code (our reanalysis used the original code, only replacing the upper limit for number of species)

Our study was motivated by concerns about unrealistically high estimates of total richness expressed to us by model users. Indeed, we find examples in the published literature where the estimated *N* is substantially larger than the number of species known to occur in the area. For instance, Loos et al. (2015) found unrealistically high estimates for butterflies and plants in a study in Romania and conducted further analyses using observed richness. Yamaura, Connor, et al. (2016) obtained an unrealistic estimate for birds in Japan and continued analyses considering only species detected at least once. In analyses of fish species in Colorado, Broms, Hooten, and Fitzpatrick (2016) found that their estimate of *N* always lied at the upper bound allowed during estimation. Our reanalysis of the bog ant data in Dorazio, Gotelli, and Ellison (2011) without constraints on *N* yields substantial support for much greater richness than expected by experts (Figure 1). Here, we seek to gain understanding about whether the method has indeed a tendency for overestimation and, more generally, learn about its estimation performance, including the impact of choice of priors in a Bayesian analysis. We also aim to address confusion about what the estimated *N* represents, which we have repeatedly encountered in discussions with model users. The concept of “total number of species” can be elusive when estimating richness, as its scope is only implicitly defined. In the context of “single‐site” estimators, O'Hara (2005) comments on the informal definition of the community and what richness estimates reflect. Similar considerations apply in the multispecies occupancy–detection modeling framework.

We organize our paper into two main parts. First, we review model assumptions, with a focus on those related to sampling, to examine what the estimated *N* represents and identify associated potential sources of bias in its estimation. Second, we explore the performance of the estimator using simulated data under a range of conditions, including ideal scenarios (data generation matching model structure perfectly) and violations of model assumptions about species heterogeneity. We assess the sensitivity of estimates to the choice of prior distributions. We complement our assessment with analyses of a real dataset on Swiss birds.

## 2 MODELING APPROACH AND INTERPRETATION OF ESTIMATES

### 2.1 Multispecies occupancy–detection model

The model is constructed with single‐species occupancy–detection models as building blocks (Dorazio & Royle, 2005; Guillera‐Arroita, 2017; MacKenzie et al., 2002). The data required are species detection/non‐detection records at a set of sampling sites, with replication such that data inform about species detectability. This replication often takes the form of repeat visits to sites (but sometimes is achieved through different means, for example, simultaneous independent observers). For each species *k*, presence or absence at site *i* is described as the outcome of a Bernoulli trial with occupancy probability *ψ_ik_*, which can be related to environmental predictors, for example, using a logistic regression:

$$z_{\mathrm{ik}}∼\;\text{Bernoulli}\;\left(\psi_{\mathrm{ik}}\right),$$

$$\text{logit}\left(\psi_{\mathrm{ik}}\right)\;=\beta_{0k}+\beta_{1k}X_{1i}+\;\beta_{\text{2}k}X_{\text{2}i}+\cdots .$$

The observed species detection/non‐detection data (*y_ijk_*), are described as another set of Bernoulli outcomes, with (detection) probability *p_ijk_* at occupied sites, where *j* refers to survey visit; this probability can be related to spatial and/or temporal predictors, for example:

$$y_{\mathrm{ijk}}∼\;\text{Bernoulli}\;\left(z_{\mathrm{ik}}p_{\mathrm{ijk}}\right),$$

$$\text{logit}\left(p_{\mathrm{ijk}}\right)\;=\alpha_{0k}+\alpha_{1k}Y_{1ij}+\alpha_{2k}Y_{2ij}+\cdots .$$

Assuming no false positives, only zeros can be recorded for a species at sites where it is absent, hence the multiplication by *z_ik_* above, in the probability of the Bernoulli. The estimated presence/absence indicators (latent variables *z*'s) and occupancy probabilities (*ψ*’s) for individual species can be used to derive estimates of diversity metrics (Broms et al., 2015; Dorazio et al., 2011; Dorazio, Royle, Söderström, & Glimskär, 2006). For instance, site‐specific predictions of richness can be obtained by computing the expected number of species at each site *i*, that is, the sum of estimated occupancy probabilities: ${\hat{N}}_{i}=\sum_{k}{\hat{\psi }}_{\mathrm{ik}}$. Predictions *conditional* on the data observed at surveyed sites can be computed by summing the latent binary presence–absence indicators: ${\hat{N}}_{i}^{\text{cond}}=\sum_{k}{\hat{z}}_{\mathrm{ik}}$ (see Kéry & Royle, 2016, pp. 569–571, for an explanation of the connection between *z*'s and conditional occupancy probabilities).

The multispecies occupancy–detection model may be simply the collection of fully independent single‐species occupancy–detection models. Alternatively, species models may be linked by modeling parameters as species random effects (Dorazio & Royle, 2005; Dorazio et al., 2006; Kéry & Royle, 2009). The latter usually takes the form of realizations from a normal distribution.

$$\beta_{0k}∼N\left(\mu_{\beta_{0}},\sigma_{\beta_{0}}\right),\beta_{1k}∼N\left(\mu_{\beta_{1}},\sigma_{\beta_{1}}\right),\ldots ,$$

where (hyper)parameters (*µ*’s and *σ*’s) are to be estimated. Through such model structure, species with fewer data “borrow” information from other species that are data‐rich, which can lead to improved precision and predictive ability (Ovaskainen & Soininen, 2011; Zipkin, DeWan, & Royle, 2009). The linking of species via random effects is also the key for making inference about the total number of species (*N*) that are present in the region of inference (i.e., including those not recorded in any visit to any of the sites). Dorazio and Royle (2005) and Dorazio et al. (2006) were the first to provide methods to tackle this estimation task. Royle, Dorazio, and Link (2007) propose to use “data augmentation,” which allows simple implementation in popular Bayesian modeling tools such as BUGS or JAGS (Lunn, Spiegelhalter, Thomas, & Best, 2009; Plummer, 2003). This is the approach we consider in this paper and involves augmenting the dataset with an arbitrary number of “potential species” with all‐zero detections, so that it contains *M* species in total (i.e.*,* detected plus potential). The choice of *M* is not critical, only that, in order to represent a vague prior on *N*; it should be made large enough not to constrain estimation, *that is,* *M* ≫ *N* where *N* is the true (unobserved) species richness (not *n*, the observed species richness). A set of binary indicators (*w_k_*) are introduced in the model, one for each of the *M* species, representing whether it belongs or not to the community. A new parameter (Ω) governs these indicators, describing the probability of species inclusion. The model structure changes slightly so that, for a species to be present at site, it first has to be a member of the community:

$$w_{k}∼\;\text{Bernoulli}\left(\Omega \right),$$

$$z_{\mathrm{ik}}∼\text{Bernoulli}\left(w_{k}\psi_{\mathrm{ik}}\right).$$

To complete the model, we define priors for the hyperparameters (*µ*’s and *σ*’s), and for the probability of inclusion (Ω). The estimation of the *total* number of species in the community (*N*) is achieved by simply summing the new indicators: $\hat{N}=\sum_{k}{\hat{w}}_{k}$. A species can thus be part of the community despite not being present at the sampled sites.

**Figure 2:**
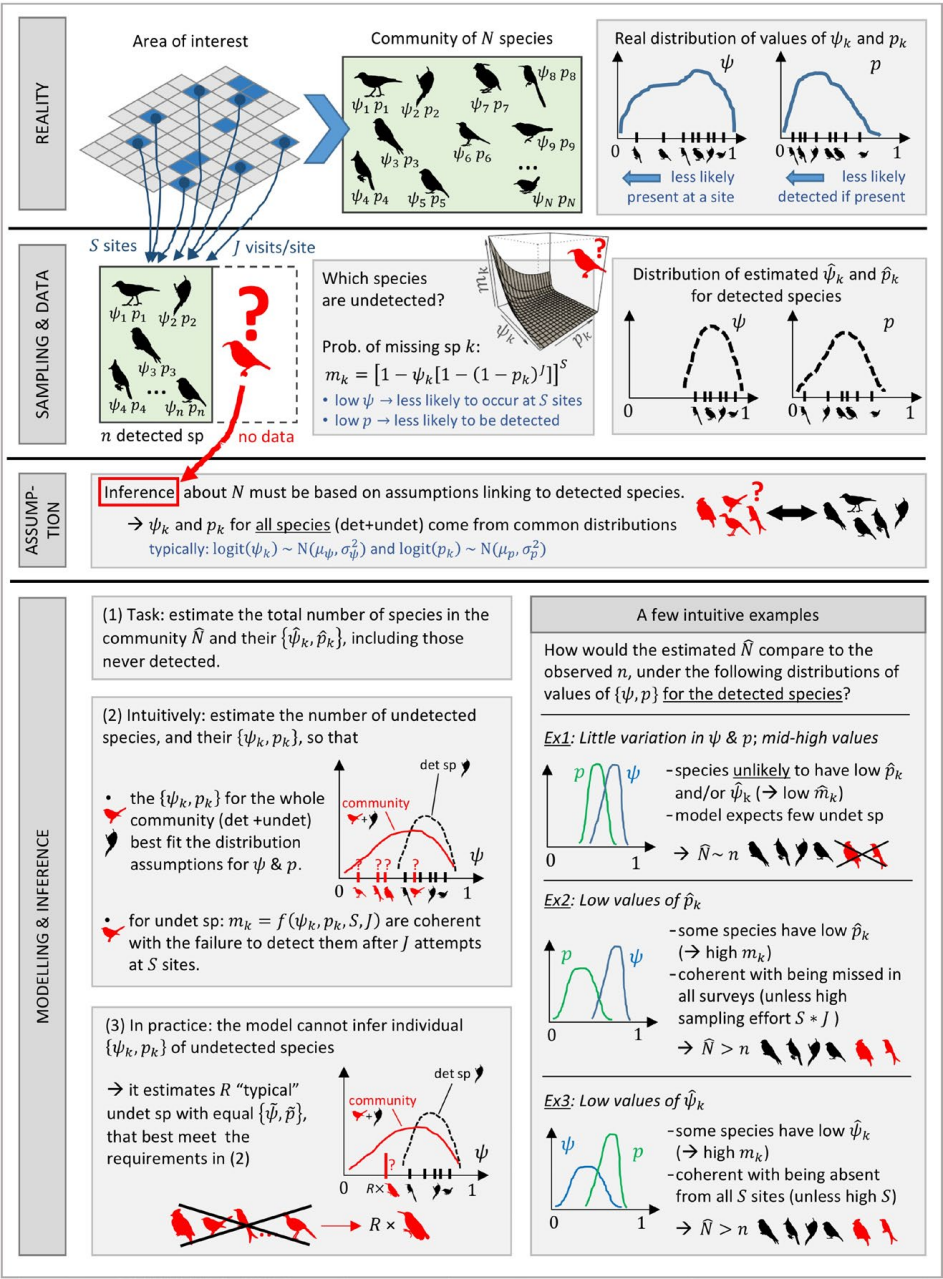
Non‐technical explanation of the estimation task (for model without predictors)

Put in simple terms, we can interpret model fitting as a process of finding statistical distributions of the specified parametric form (usually, normal random effects) that best fit the occupancy and detection parameters estimated for the species detected, while considering the possibility of “adding” a number of undetected species to the pool (Figure 2).

### 2.2 Interpretation of N and sampling considerations

The interpretation of *N* is often not evident for users of hierarchical occupancy models. This is because the definition of what constitutes the community whose size is estimated is set implicitly by the characteristics of the sampling and corresponding model assumptions. If the user inadvertently overlooks these assumptions, the quantity estimated by their model may not reflect what they were actually seeking to estimate, and apparent biases could be observed.

Two important considerations relate to the sampling of sites. First, there is an assumption of random sampling behind the computation of *N*, with the region selected for sampling providing the spatial context needed to interpret *N* (Dorazio et al., 2006). For instance, if we draw survey sites at random across Switzerland, then *N* refers to the number of species in Switzerland (but note an important further consideration below). In contrast, *N* is ill‐defined if sites are sampled such that some environmental conditions (e.g., low elevation) are disproportionally represented. When sampling is biased, we can think of *N* as the number of species that would be present in a large hypothetical region with environmental conditions in the proportions captured by the sample of surveyed sites. Another consideration is that this is an asymptotic approach which assumes the region from which sampling sites are drawn is “very large” (i.e., contains an infinite number of potential sites; c.f. Dupuis & Goulard, 2011). If departure from this assumption is substantial, and the region of inference is relatively small, then *N* may overestimate the number of species; this is because the chances that a species is present in at least one site are higher the larger the pool of sites considered. Departures from these two assumptions may be addressed by, rather than focusing on the estimated *N*, computing new richness predictions based on the predicted occurrence status (*z*) for all species only at the set of sites that defines the actual region of inference (note this will generally extend beyond the specific set of sites sampled); in practice, this involves counting the number of species with at least one latent site occupancy binary indicator estimated equal to 1 in the set of sites making up the target region of inference (Kéry & Royle, 2009).

The definition of *N* also depends critically on considerations about sampling of species; it only encompasses the set of species that were susceptible to detection. Data on insects will not yield predictions about number of bird species. Similarly, *N* will not reflect true bird richness if surveys only targeted a subset of bird species. One simplistic way to think about this is that the model infers the likelihood that species are missed (i.e., a proportion) and “corrects” the number of species detected by that proportion. If we start with a count lower that the true number we would have observed, the resulting estimate will be biased low. Bias will be also induced if the sampling methods render some species virtually undetectable (e.g., nocturnal species during day surveys).

These sampling considerations fundamentally define *N*. Another issue is how well this quantity can be estimated in practice. The model involves other assumptions (e.g., distribution of species random effects) and estimates will be to some degree sensitive to violations of these assumptions. A yet more basic question is how accurate estimation is when all assumptions are perfectly met. We concentrate on exploring these issues in the remainder of the paper.

## 3 METHODS: SIMULATION AND DATA ANALYSES

We used simulated and real data to explore the performance of the richness estimator, as detailed below. We ran analyses in R v3.3.1 (R Core Team, 2016), conducting Bayesian model fitting in JAGS v4.2.0 (Plummer, 2003), interfaced through package jagsUI 1.4.4 (Kellner, 2016). We provide our code as Supporting Information.

### 3.1 Estimator performance under ideal conditions

We start by considering analyses under ideal conditions, meaning that the data generating process matches perfectly the model assumptions. This sets an upper bound for estimation performance. We simulated the sampling of a community of 100 species over a large landscape (consisting of 10,000 sites). We considered each species *k* to have different occupancy and detectability probabilities, constant across the landscape (for the sake of simplicity) and drawn from a common distribution. We used two scenarios of occupancy probabilities:

$$\text{logit}\left(\psi_{k}\right)=\beta_{0k}∼N\left(\mu =-1,\sigma =0.3\right)\text{Scenario ``Occ1''},]]>$$

$$\text{logit}\left(\psi_{k}\right)=\beta_{0k}∼N\left(\mu =-2,\sigma =0.6\right)\text{Scenario ``Occ2''}.]]>$$

Scenario “Occ1” corresponds to a mean occupancy probability of 0.27, with values in the range [0.17–0.40] for 95% of the simulated species. In “Occ2”, occupancy probabilities have mean 0.13 and range [0.04–0.30]. We considered that species detection probabilities follow logit(*p_k_*) ~ *N*(−2,1), so that the mean probability of detecting a species in one survey visit is 0.15, and within [0.02–0.49] for 95% of the species. We simulated nine sampling regimes, with different combinations of number of randomly sampled sites (*S* = 25,50,150) and survey visits per site (*J* = 2,4,6). The probability of completely missing a species *k* (i.e., not a single detection after *J* visits at *S* sites) is $m_{k}={\left(1-\psi_{k}p_{k}^{*}\right)}^{S}$, where $p_{k}^{*}=1-{\left(1-p_{k}\right)}^{J}$ is the cumulative probability of detection at occupied sites over the *J* visits, that is, $m_{k}={\left[1-\psi_{k}\left\{1-{\left(1-p_{k}\right)}^{J}\right\}\right]}^{S}$. With our choice of parameters, we tested the method under conditions that range from a substantial number of species missed in sampling, to cases where all species are recorded at least once (Table 1). We wanted to explore how good the method is at estimating number of missing species across this spectrum, including at establishing that no species are missed when this is the case. Our choice of simulation parameters is in line with those used in a previous simulation study based on values estimated in the literature (Broms et al., 2015).

**Table 1:** Expected proportion of species missed in each simulated scenario (in brackets, the proportion of species in the simulated community not present in any of the sites sampled)

	Scenario “Occ1”			Scenario “Occ2”		
	*S* = 25	*S* = 50	*S* = 150	*S* = 25	*S* = 50	*S* = 150
*J* = 2	27% (0%)	13% (0%)	2% (0%)	50% (8%)	32% (2%)	10% (0%)
*J* = 4	14% (0%)	5% (0%)	1% (0%)	35% (8%)	19% (2%)	4% (0%)
*J* = 6	9% (0%)	3% (0%)	0% (0%)	27% (8%)	13% (2%)	2% (0%)

Note

*S* is the number of sites sampled, *J* the number of survey visits per site. In “Occ1,” species occupancy probabilities are normally distributed on the logit scale as *N*(−1,0.3); in “Occ2” as *N*(−2,0.6). In all cases, detection probabilities in the logit scale follow *N*(−2,1).

We simulated and analyzed ten datasets for each of the scenarios of occupancy and sampling regime (2 × 9 × 10 = 180 datasets in total). The fitted model assumed constant occupancy and detection probabilities within species (i.e., no predictors, matching our data generation). We augmented the datasets by adding a number of potential “undetected species” (with all‐zero records), from 50 up to 500, depending on the dataset. We chose the number by running analyses with increasing number of species, until the posterior of *N* was not affected by this decision, or at least not substantially. We assessed this by dividing the interval between the lowest value sampled for *N* and the maximum number of species allowed (*M*) into 10 sections of equal width. We considered data augmentation satisfactory if the posterior mass within the upper section was <1%. For practical reasons, we limited the number of added “species” to 500.

We analyzed each simulated dataset using three sets of priors (resulting in 180 × 3 = 540 analyses), representing common practice and recommended alternatives (see Section 3.4). We obtained 3 MCMC chains, drawing 50,000 MCMC samples per chain, after a burn‐in of 25,000. If chains had not converged, additional samples were drawn in blocks of 50,000, up to a maximum of 200,000; we only kept the last block of 50,000 samples, discarding others as burn‐in. We assessed convergence using the $\hat{R}$ statistic (Gelman & Rubin, 1992), assuming no evidence of lack of convergence when $\hat{R}$ < 1.1. To avoid the need to save large files, we thinned chains by 25, keeping a total of 6,000 samples (i.e., 2,000 per chain) to characterize the posterior. We initialized the latent occupancy state of sites to the observed detections, and all augmented species as absent from the community (i.e., *w* = 0). For each simulation, we kept track of the number of species present at least once in the set of sites sampled, and the number of species detected by sampling. We computed summaries of the posterior of *N* (median, 2.5% and 97.5% quantiles).

### 3.2 Impact of violations of species random effects assumptions

We ran a second set of simulations to explore how violations of assumptions about the distribution governing species random effects may affect the estimation of *N*. We focused on the distribution of detection probabilities; similar tests could be run for the occupancy component of the model. The simulation set up was largely as above: 100 species, random sampling, occupancy scenarios “Occ1”/”Occ2”. The difference was in how we generated the detection probabilities when simulating the data. Rather than using a normal distribution on the logit scale, as customarily assumed during model fitting, we considered five other distributions, including with fatter or steeper tails, and/or more than one mode (details in Supporting Information Appendix S4). We focused this exploration in scenarios where relatively few species are missed (around 2% and 9%) because these are conditions yielding relatively accurate estimation in an ideal setting (see Section 4) and we wanted to assess how robust estimation was to these deviations. In all cases, we set the number of sampling sites to 25 and designed the detectability scenarios to meet the chosen level of missed species (alternatively, one could build scenarios with more sampling sites and lower detectabilities). We analyzed 10 simulated datasets per scenario. Fitted models assumed a normal distribution for the description of random effects. We augmented the data set by 500 additional species and used the same MCMC settings and priors as above.

### 3.3 Swiss bird data

Our third set of analyses were based on a real dataset, which we resampled to simulate sampling scenarios with different amounts of data. Our aim here was to explore estimator performance under realistic conditions and compare estimates for different amounts of data and structure of analysis. The data were collected as part of the Swiss breeding bird survey (MHB; Schmid, Zbinden, & Keller, 2004), in which all birds breeding in Switzerland are surveyed annually in 267 1 km^2^ quadrats laid out as a grid. Three surveys (two in sites at high elevation) are conducted per site. The dataset is publicly available with R package “AHMbook” (companion of Kéry & Royle, 2016). It contains records collected in 2004 (detection/non‐detection, reduced from the original survey counts) for 158 species (of which 15 had no detections that year). Starting with the full dataset, we created 16 new datasets by randomly partitioning the sites into groups with 50% (two datasets), 25% (four datasets), or 10% (10 datasets) of the sites. We analyzed the datasets (original plus subsets) fitting two models: (a) with constant occupancy and detectability; and (b) with predictors. For the latter, we used the structure in Kéry and Royle (2016, pp. 685–694), where occupancy is a function of elevation (quadratic) and forest cover, and detectability is a function of survey date (quadratic) and survey duration; here species random effects are on regression coefficients. With real data in principle, we do not know the true number of species. However, as Swiss birds have been thoroughly studied, there is a good understanding about the number of breeding bird species in the country (179 regular, 20 irregular, 16 occasional; Kéry & Royle, 2016, p. 689), which we can use as a benchmark to compare our estimation against. Analyses were conducted drawing three MCMC chains, with 20,000 MCMC samples per chain, after a burn‐in of 10,000. If there was no convergence, 20,000 additional samples were drawn, with all previous ones discarded as burn‐in. We thinned chains by 10, obtaining 6,000 samples to characterize the posterior. Datasets were augmented with 150 (models without predictors) or 250 (models with predictors) additional all‐zero species; thus, the maximum total number of species allowed was 308 and 408, respectively. Predictors were standardized to have zero mean and variance of 1. As with simulations, we analyzed each dataset using three sets of priors (detailed next).

### 3.4 Choice of priors

We ran our analyses assuming no prior information about model parameters, and therefore aimed to be weakly informative with our choice of priors. We considered three sets. Our “prior set 1” followed common practice. For the means of hyperdistributions (the *µ*’s), we used wide normal priors (with standard deviation of 31, i.e.*,* precision 0.001). Our literature review (Table S.1.1) showed this was a common choice (over half the studies that estimated *N* used priors at least as wide for the mean of regression coefficients). For the standard deviation of the hyperdistributions (the *σ*’s), we used uniform priors with support in the [0–5] range (for the Swiss data analyses, we increased the range to [0–10] in one instance). These were a common choice in the studies we reviewed; gamma priors were also common. For Ω, we used a uniform in 0–1 (used by all but one of the studies reviewed), which implies a discrete uniform prior for *N* on {0, 1, 2, … *M*}.

In “prior set 2,” we modified the priors for the means of the hyperdistributions. It has been recommended that priors for logit‐scale parameters should have low mass outside the range [−5, 5] (Broms et al., 2016; Gelman et al., 2014). Otherwise, estimation can be biased toward extreme probabilities values (0, 1), because a wide prior on the logit scale results in a U‐shaped prior for associated probabilities. Following this advice, we used narrower normal priors for the µ’s (*N*(0,2.25)).

In “prior set 3,” we made a further modification to “prior set 2” and replaced the uniform prior for Ω by a Beta(0.001,1), following recommendations by Link (2013), who argues strongly against the use of the constant prior due to the potential for it to yield improper posterior distributions.

## 4 RESULTS

### 4.1 Estimator performance under ideal conditions

**Figure 3:**
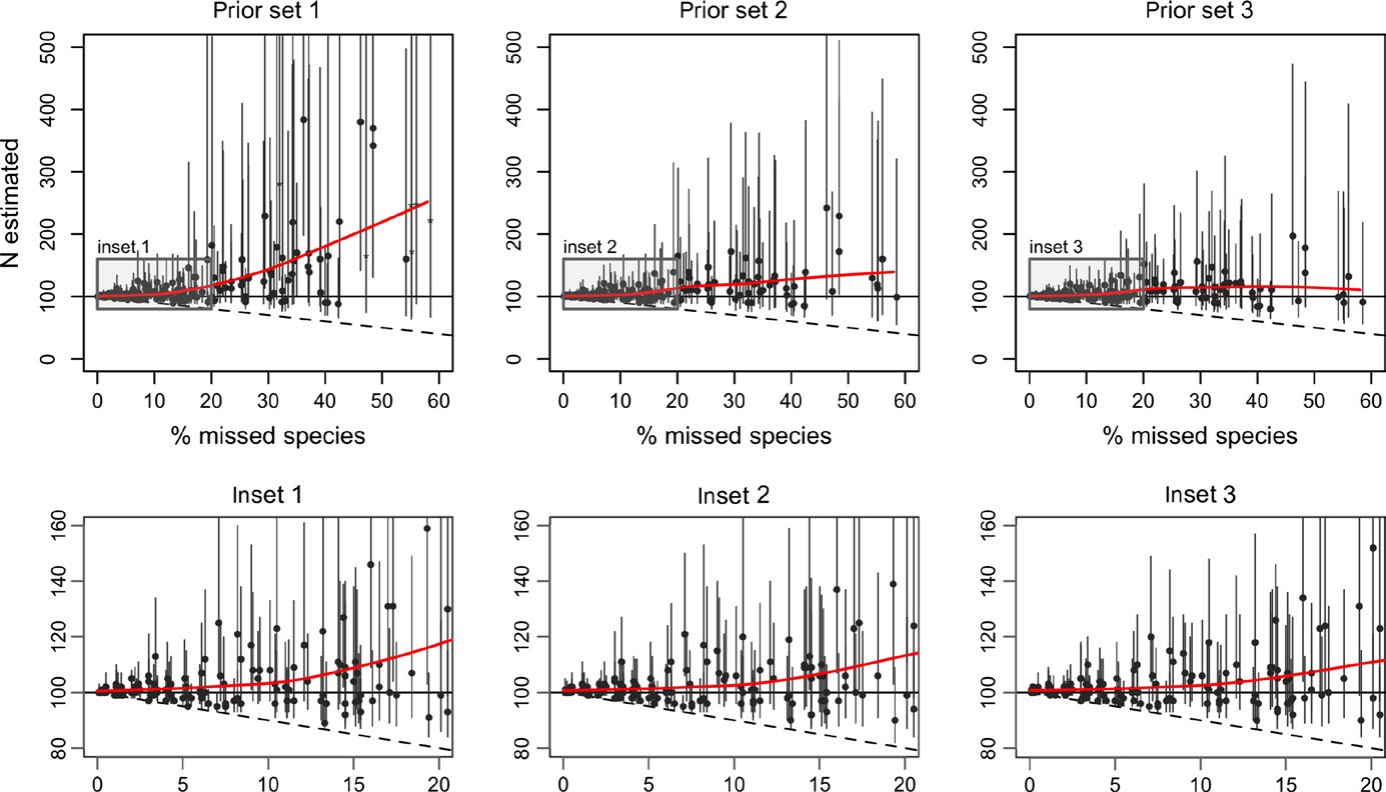
Estimated number of species (N) as a function of the percentage of species missed during sampling, for 180 simulated datasets generated meeting perfectly model assumptions, fitted using three different sets of priors (columns). Bottom row: close up of the boxed area in the main plots (top). Markers indicate the posterior median; lines represent equal‐tailed 95% credible intervals. The solid horizontal line corresponds to the true number of species (100), and the dashed line indicates the number of species detected during sampling. The red solid line is a smoother applied to the medians, to indicate bias. In the top left figure, small markers (stars instead of circles) indicate that the level of data augmentation chosen influenced the posterior. Simulated scenarios as detailed in Table 1 (10 simulations/scenario)

In our simulations under ideal conditions, the estimation of *N* was relatively reliable when few species were missed; however, it was poor when the proportion of species missed was substantial (Figure 3 and Supporting Information Appendix S2), suggesting a tendency for overestimation. Results show a positive correlation between departure of the posterior median from the truth, and the width of credible intervals, with much greater uncertainties when *N* is overestimated.

Overestimation was greatest with “prior set 1,” which represents usual practice, with the posterior distribution for *N* often extending well beyond the true number of species. In 34 of the 180 simulations, the 95% credible interval for *N* spanned all the way to 300 species (three times more than the simulated “truth”), going over 500 species in 19 simulations. These more extreme estimates corresponded to cases with a substantial number of missed species (range: 19–58, mean = 40), and the maximum level of data augmentation allowed (+500 species) was still not enough to avoid constraining the posterior of *N*; even greater estimates could be expected by relaxing this limit. Estimates were less extreme when we used the alternative sets of priors (Figure 3). Narrowing down the priors of the logit scale parameters (“prior set 2”) had the strongest effect in this reduction. Then, the credible interval spanned beyond 300 in only 18 simulations, and over 500 in just two. The scale prior recommended by Link (2013) (“prior set 3”) improved accuracy further, with only five simulations spanning over 300 and none over 500.

A look at the convergence metrics for “prior set 3” reveals that values of $\hat{R}$ suggestive of lack of convergence (>1.1) remained for some simulations (*n* = 49, out of 180), even after increasing the number of MCMC samples to 200,000. Large $\hat{R}$ values were usually on the community inclusion binary indicators (the *w*'s), or on *N*. $\hat{R}$ values for the occupancy and detection hyperparameters were >1.1 in only 19 simulations, and over 1.2 in just 7. In contrast, in a substantial number of simulations (*n* = 28), $\hat{R}$ for *N* was very large (>1.5, up to “infinity”). Interestingly, this happened in scenarios where very few or none of the species were missed. Inspection of the MCMC chains revealed that these large $\hat{R}$ values corresponded to very slow mixing of the binary indicator variables, and consequently of *N* (see Supporting Information Appendix S3 for an example). Such slow mixing did not happen in scenarios with fewer data, or lower probabilities of occupancy and detection, where species were missed more often during the surveys.

### 4.2 Impact of parametric assumption violations

**Figure 4:**
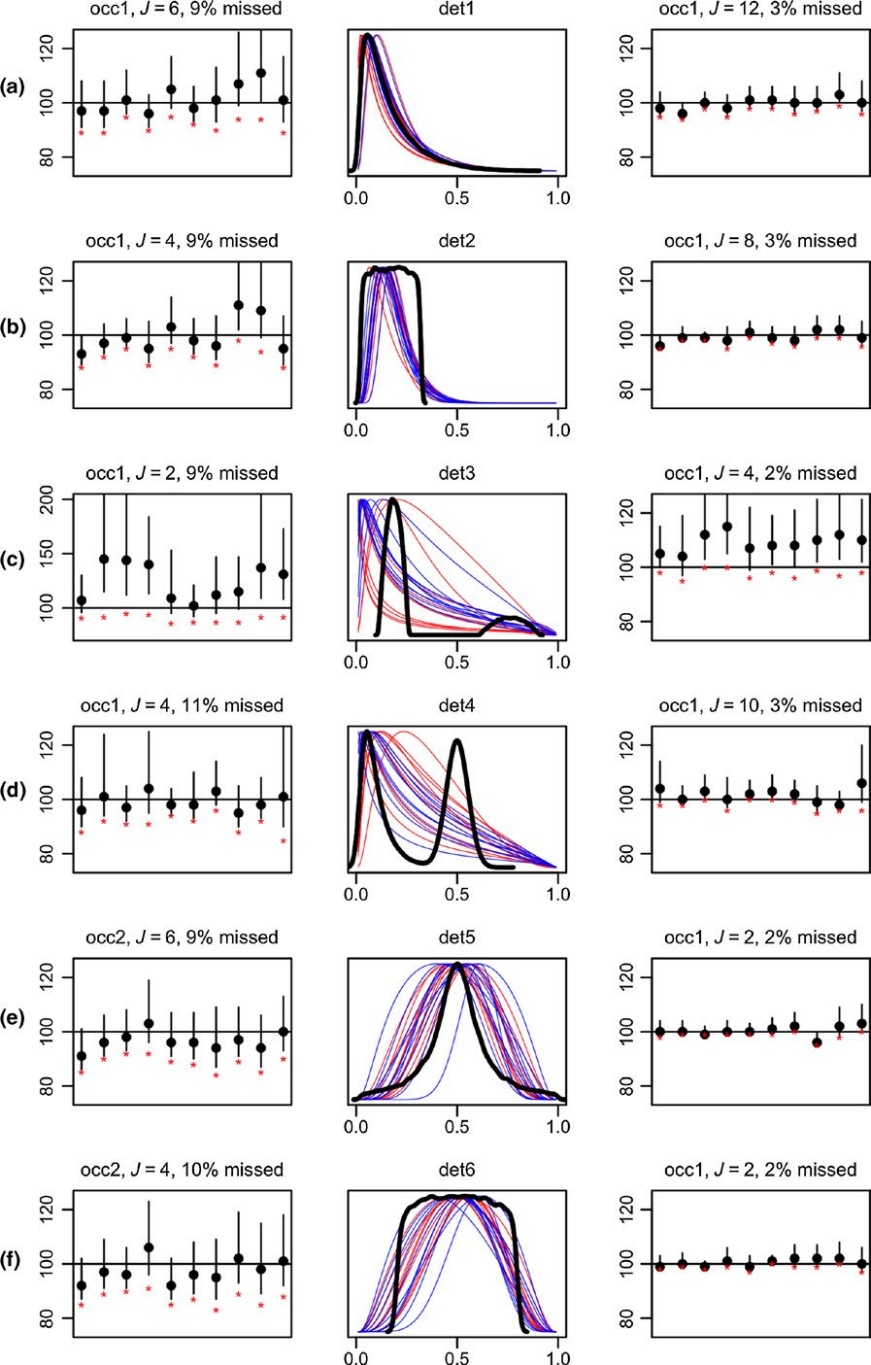
Estimation under violation of parametric assumptions about species detectability, for scenarios with small proportion of species missed. Plots in columns 1 and 3 show the number of species estimated for 10 simulations, under the detectability scenario described by the probability function in the middle column (thick black line). Plot titles indicate the occupancy scenario (“occ1”/”occ2”) and number of replicates (*J*) used. The number of species missed in these scenarios was around 10% (left column) or 2%–3% (right column). In all cases the number of survey sites *S* was 25, and prior set 3 was used. Red stars indicate the number of species detected during sampling for each dataset. The thin colored lines in the middle plots are the community normal distributions estimated in each simulation run (red: left column; blue: right column)

Simulation results were similar across the three sets of priors; we only report here results for “prior set 3.” The assumption violations about species random effects on detectability had variable effects on the estimation of *N* in our scenarios. Substantial deviations from the normal distribution assumption often did not introduce obvious performance degradation (Figure 4). However, there was a substantial effect in one scenario where the generating distribution displayed bimodality with the first mode away from zero (Figure 4c): prediction was much more uncertain than in the other examples (note different y axis in one of the plots), and there was substantial overestimation of *N*. The normal distributions that best fitted the variation in detectability had substantial mass close to zero, unlike the distribution used to generate these probabilities. Hence the model concluded that many more species had been missed than the actual number missed.

### 4.3 Swiss bird data

**Figure 5:**
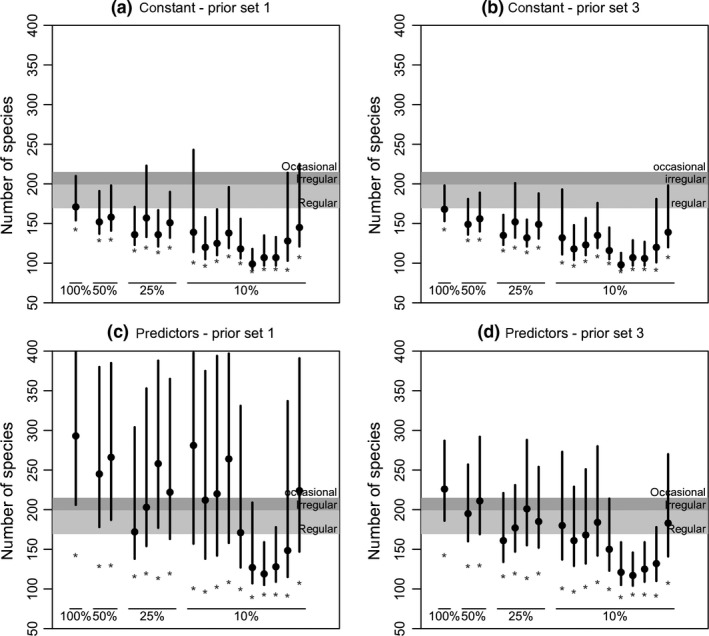
Estimated number of breeding bird species in Switzerland, obtained analyzing the full 2004 MHB dataset (267 sampling sites) and subsets (50%, 25%, 10% of sites), for two sets of priors (1 and 3). In (a, b) no predictors were used, and in (c, d) predictors were included both in occupancy and detection probabilities (following Kéry & Royle, 2016, pp. 685–694). Dots show the median of the posterior distribution, and lines are equal‐tailed 95% credible intervals. Reference levels show the number of bird species known to breed in Switzerland regularly (179), irregularly (+20), and occasionally (+16). Stars indicate the number of species detected in each data set. The maximum number of species allowed in analyses were (a, b) 308 and (c, d) 408

The results of the Swiss bird analyses displayed considerable variation in the estimation of *N*. Results were relatively precise for constant models but suggested a tendency for underestimation, particularly for smaller sample sizes (Figure 5a,b). For more complex models with predictors in occupancy and detectability, estimates became more variable and uncertain (Figure 5c,d), more so with “prior set 1.” Several analyses yielded median predictions larger than the number of birds known to breed in Switzerland, with credible intervals extending to substantially larger numbers (although in general also covering the numbers expected by experts). Still, a few analyses yielded likely underestimates, which were deemed relatively precise by the model. We found substantial differences between analyses of one same dataset with and without predictors, sometimes with little overlap in credible intervals. For instance, in one of the subsets with 25% of data (third of 25% in Figure 5a–d) and “prior set 1,” the constant model suggested the number of breeding bird species to be between 121 and 184 (median 136), while the model with predictors suggested a number between 170 and 397 (median 263). The maximum $\hat{R}$ was greater than 1.1 for some of the analyses (7 with prior set 1; only 2 with prior set 3), and in all cases was less that 1.25.

## 5 DISCUSSION

Multispecies occupancy–detection modeling allows inference about the number of species completely missed by sampling in spatially and temporally replicated surveys. It is important to understand the sampling assumptions underlying the method, as these fundamentally determine the interpretation of the estimated quantity. We have explored the performance of this estimation task in a range of scenarios, with the aim of informing ecologists planning to put this method into practice. We tested the method using JAGS (Plummer, 2003) for model fitting because, together with BUGS (Lunn et al., 2009), this is the tool of choice for most users. Our study is not without limitations. The spectrum of scenarios tested, although substantial, represents only a small fraction of the many one could devise. Also, as model fitting is computationally intensive and can take substantial time depending on the scenario, we ran a modest number of repetitions (10) per scenario. With this amount of replication, we cannot quantify precisely estimation bias and precision per individual scenario. Yet, taken *across* simulated scenarios, we believe our results are comprehensive enough to provide a good indication of general performance patterns.

Our study was motivated by concerns about unrealistic richness estimates expressed by model users. Indeed, our simulations suggested that unrealistically high estimates with large uncertainty may often be obtained when the number of species missed is moderately high (~15% or more), even when all model assumptions are met. We therefore suggest that large estimates with broad credible intervals should be interpreted as an indication that species might have been missed, rather than as a reliable indication of species numbers. Where many species are missed, the sample size and/or the occupancy/detection probabilities are small. These are conditions that do not allow reliable estimation of occupancy and detectability, so it is unsurprising that the associated estimation of *N* is also poor. This interpretation extends to analyses where the posterior of *N* remains constrained despite increasing the amount of data augmentation, hence suggesting increasingly larger richness estimates. Large uncertainty when many species are missed has been reported previously for other richness estimation methods (e.g., Gotelli & Ellison, 2012, p. 474). We found the choice of priors to have a strong effect on the tendency for overestimation, with priors that are commonly used in these studies (broad priors on the logit scale; uniform prior on *N*) accentuating the problem. We therefore suggest assessing the sensitivity of estimates to prior choice, considering priors that follow best practice recommendations (as in our prior set 3, and in Broms et al., 2015). Also, where prior information on the maximum number of potential species is available, this can be incorporated as a constraint on *N* (by setting *M* accordingly; e.g., Dorazio, Kéry, Royle, & Plattner, 2010; bog ant study of Dorazio et al., 2011), mitigating thus the risk for overestimation.

Looking at the results of our ideal simulations, one could conclude that the method is fairly reliable in scenarios with a small proportion of missed species, yielding estimates not far from truth and with relatively narrow credible intervals. This would suggest that users can take precise estimation around the number of detected species as a likely indication that no or few species have been missed in their surveys. However, the risk exists that such precise estimates are actually the result of underestimation. Departures from model assumptions could lead to such situations. For instance, our analyses of a real dataset (Swiss birds) showed estimates of number of species to be quite sensitive to model structure, with an apparent tendency for underestimation when relevant predictors were not included in the model (also observed by Kéry & Royle, 2016).

The distribution of the species random effects describing occupancy and detectability is key assumptions in the model. Our simulations breaking the normality assumption about detection probabilities highlighted that departures in the lower tails of the distributions are the most problematic. This is because the lower tail indicates the frequencies of less detectable species (or for occupancy, the less prevalent), that is, those most likely to be missed. If the mass of the lower tail is overestimated, the number of species can be substantially overestimated. This can happen even with thorough sampling and few missed species, as in our simulated examples. It cannot therefore be assumed that estimation is generally reliable when operating in scenarios with few missed species (unlike suggested by the ideal simulations). These results also indicate that estimates suggesting a greater number of species than detected may just be the product of model assumption violations. Conversely, richness will be underestimated if the mass in the lower tail is underestimated. All of this highlights the value of evaluating fit to model assumptions, and in particular to the lower tails of the fitted distributions. Work developing and testing appropriate goodness‐of‐fit tests in this context would therefore be valuable. However, there is a limit to the help these tests can offer, as there is always the risk that the assumed parametric form fits well the data for the observed species but its lower tail fails to reflect the unobserved truth about the species missed (e.g., if there is a group of very elusive species, much harder to detect that the rest).

Apart from the performance issues above, another consideration when deciding to extend the multispecies occupancy–detection model for estimating total richness is the associated increase in computational burden. Allowing for missing species through data augmentation slows down model fitting substantially, particularly when adding many all‐zero species. The number of estimated parameters increases, and more samples are required to achieve convergence. For instance, our analyses of the full Swiss dataset with covariates took around 35 hr with prior set 1, 30 hr with set 2 and 20 hr with set 3; in contrast, model fitting restricted to the observed species and without estimation of total richness took <7 hr (Dual Intel Xeon E5‐2699 server, 2.30 GHz, ~40 GB RAM).

Any statistical modeling method can be “broken” by testing it under scenarios that violate its assumptions; the more interesting question is how *sensitive* it is to likely moderate violations. Our results demonstrate that inference about total richness from multispecies occupancy–detection models can be strongly affected by model structure (including prior choice, parametric assumptions, and inclusion of predictors). Appropriate selection of priors, testing of assumptions, and model refinement are therefore all important to enhance reliability of estimation. Nevertheless, even assuming ideal conditions, our results confirm that richness estimates may easily become unrealistically high. The inference task about *N* is inherently difficult, as it requires extracting conclusions about species that have never been observed. The reliability of other richness estimation methods has been previously seriously questioned, with the concern that it is impossible to know how bad estimates are (O'Hara, 2005). Similarly, here we argue that expectations about richness prediction accuracy should be kept realistic. We cannot model our way out of every situation. Hence, where accurate knowledge about total number of species is considered truly critical for management or policy, survey effort should ideally be such that the chances of missing species are low (so our raw data already provides a relatively good representation of species numbers). With this, we do not imply that smaller datasets are not useful, as at the very least they can yield key information about the pool of observed species. Indeed, an important fundamental consideration before data collection and analysis is whether knowledge of *N* is critical for one's application, or whether focus can be on a species pool defined a priori (e.g., "all species of taxon *X* ever observed in region *Y*").

## CONFLICT OF INTEREST

None declared.

## AUTHOR CONTRIBUTION

GGA conceived the idea; GGA and JJLM designed the study, with input from MK; GGA conducted simulations and analyses; all authors contributed to interpretation of results; GGA led the writing of the manuscript; all authors contributed to the drafts and gave final approval for publication.

## Data Availability

Our simulated datasets are reproducible from our code. The Swiss dataset is available in R package “AHMbook.”
