# Supplementary material for: Inferring species richness using multispecies occupancy modeling: Estimation performance and interpretation
Source: Ecol Evol. 2019 Feb 5;9(2):780–92. doi: 10.1002/ece3.4821 (PMC6362448; doi:10.1002/ece3.4821)
Supplement: Supplementary file 1 [file ECE3-9-780-s001.zip › ece34821-sup-0001-AppendixS1.pdf]

Appendix S1 – Literature review

Methods

We conducted three searches for relevant articles (14/Sept/2016). First, we searched ISI Web of Science for peer-reviewed journal articles citing either Dorazio & Royle (2005) (S1) or Dorazio *et al.* (2006) (S2). To complement these results, ensuring coverage of more recent articles, we conducted a key word search in Google Scholar (searched terms: "species richness" AND "data augmentation" AND "imperfect detection" AND "occupancy") (S3). Of a total of 332 initial results (S1 =123, S2 = 142 and S3 = 67), 201 records were reviewed, due to the exclusion of 131 records for reasons including duplication (102), reports/thesis (9), unavailability online (16), language other than English (3) and duplication of data (1). Of the 201 reviewed records, 112 did not use multispecies occupancy-detection models (MSODMs), so they were not considered any further.

For all the papers that fitted MSODMs, we recorded information about the study location (country/region, type of habitat), main purpose of the study, taxa, species (number in the dataset, whether species groups were separately defined for analyses), sampling (how sites sampled, number of years/season, survey type, method of replication, number of sites, number of replicates).

For all the papers that included estimation of total N, we also recorded the upper bound set for N (i.e. how many species were included in the augmented dataset), the estimated N (with confidence/credible intervals were available), and any comments by the authors about the reliability of the estimation.

Our coding book is provided as a separate spreadsheet (excel file).

Key references

Dorazio, R.M. & Royle, J.A. (2005). Estimating Size and Composition of Biological Communities by Modelling the Occurrence of Species. *Journal of the American Statistical Association*, 100, 389-398

Dorazio, R.M., Royle, J.A., Söderström, B., & Glimskär, A. (2006). Estimating Species Richness and Accumulation by Modeling Species Occurrence and Detectability. *Ecology*, 87, 842-854

Highlights

- Multispecies occupancy-detection models (MSODMs) are increasingly used (Fig S1.1)
- 35% of the studies that fitted MSODMs included also inference about total N via data augmentation.
- MSODMs have been applied to a range of taxa, most frequently to birds (60% of the studies), but also to reptiles and amphibians (15%), invertebrates (15%), fish (12%), mammals (8%) and plants (4%).
- The number of species included in the studies is highly variable (mean = 75, min = 3, max =440, median=50)
- In most studies, all species are included in a single model. Where separate models were built, the criterion used for group allocation was: foraging guild (4), taxa or life form (3), habitat/locations (10), functional group (1) and threatened status (1)
- Of the studies that estimated total N, few made explicit statements about how the estimation matches expectations or other estimates. Of these, three studies reported unrealistically high estimates, including one where estimation was always at the allowed upper bound of N. Three studies reported estimates for N substantially higher than obtained with other methods.
- Of studies that estimated total N and reported priors (Table S1.1), a majority (13) used wide priors for the parameters on the logit scale (mean of hyperparameters), with a standard devia-

tion equal or greater than 31 (i.e. precision equal or smaller than 0.001). For the spread of hyperparameters, most studies used a gamma (13) or a uniform (9) distribution. For the community inclusion parameter (Ω) all but one study used a uniform from 0 to 1.

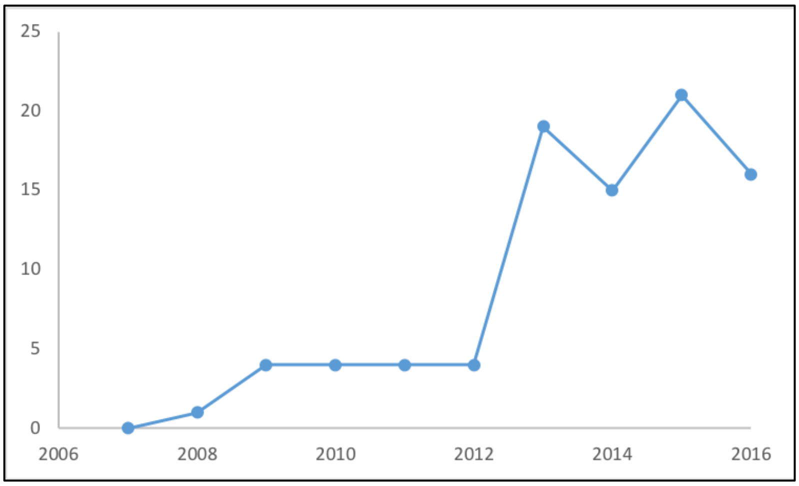

Fig S1.1: number of reviewed articles that fitted MSODMs, per publication year. Note that data for 2016 is incomplete, as this review was conducted in September 2016.

hyperparameter means	
N(0,1.5)	1
N(0,2.25)	2
N(0,3.1)	1
N(0,31)	5
N(0,100)	2
N(0,1); U(0,1) for intercept*	1
N(0,10); U(0,1) for intercept*	2
N(0,31); U(0,1) for intercept*	6
U(0,1) (only intercept*)	3

hyperparam spread	
s ~ half-Cauchy(2.25^2)	1
tau ~ Gamma (0.1,0.1)	10
tau ~ Gamma (0.01,0.01)	3
s ~ U(0,5)	2
s ~ U(0,10)	7

omega	
U(0,1)	24
beta(0.001,1)	1

Table S1.1: Priors used by the reviewed studies that estimated N via data augmentation, and number of studies using them. * priors defined on the probability scale (otherwise, on the logit scale). tau = precision; s = standard deviation

List of papers reviewed (order as in the coding book)

1. Goijman, A. P., Conroy, M. J., Bernardos, J. N. & Zaccagnini, M. E. Multi-season regional analysis of multi-species occupancy: Implications for bird conservation in agricultural lands in east-central Argentina. PLoS ONE 10, (2015).
2. Carrillo-Rubio, E. et al. Use of Multispecies Occupancy Models to Evaluate the Response of Bird Communities to Forest Degradation Associated with Logging. Conserv. Biol. 28, 1034–1044 (2014).
3. Loos, J. et al. Developing robust field survey protocols in landscape ecology: a case study on birds, plants and butterflies. Biodivers. Conserv. 24, 33–46 (2015).
4. Rich, L. N. et al. Comparing capture-recapture, mark-resight, and spatial mark-resight models for estimating puma densities via camera traps. J. Mammal. 95, 382–391 (2014).
5. Sutherland, C., Brambilla, M., Pedrini, P. & Tenan, S. A multiregion community model for inference about geographic variation in species richness. Methods Ecol. Evol. 7, 783–791 (2016).
6. White, A. M. et al. Avian community responses to post-fire forest structure: implications for fire management in mixed conifer forests. Anim. Conserv. 19, 256–264 (2016).
7. Woolley, S. N. C. et al. Deep-sea diversity patterns are shaped by energy availability. Nature 533, 393–396 (2016).
8. Latif, Q. S., Sanderlin, J. S., Saab, V. A., Block, W. M. & Dudley, J. G. Avian relationships with wildfire at two dry forest locations with different historical fire regimes. Ecosphere 7, (2016).
9. Ramesh, T., Kalle, R., Rosenlund, H. & Downs, C. T. Native habitat and protected area size matters: Preserving mammalian assemblages in the Maputaland Conservation Unit of South Africa. For. Ecol. Manag. 360, 20–29 (2016).
10. Pearson, S. F., Giovanini, J., Jones, J. E. & Kroll, A. J. Breeding bird community continues to colonize riparian buffers ten years after harvest. PLoS ONE 10, (2015).
11. McNew, L. B. & Handel, C. M. Evaluating species richness: Biased ecological inference results from spatial heterogeneity in detection probabilities. Ecol. Appl. 25, 1669–1680 (2015).
12. Flanders, N. P. et al. Key seabird areas in southern New England identified using a community occupancy model. Mar. Ecol. Prog. Ser. 533, 277–290 (2015).
13. Tobler, M. W., Zúñiga Hartley, A., Carrillo-Percastegui, S. E. & Powell, G. V. N. Spatiotemporal hierarchical modelling of species richness and occupancy using camera trap data. J. Appl. Ecol. 52, 413–421 (2015).
14. Russell, J. C., Stjernman, M., Lindström, Å. & Smith, H. G. Community occupancy before-after-control-impact (CO-BACI) analysis of hurricane gudrun on swedish forest birds. Ecol. Appl. 25, 685–694 (2015).
15. Gilroy, J. J. et al. Minimizing the biodiversity impact of Neotropical oil palm development. Glob. Change Biol. 21, 1531–1540 (2015).
16. Lewis, T. L., Lindberg, M. S., Schmutz, J. A., Bertram, M. R. & Dubour, A. J. Species richness and distributions of boreal waterbird broods in relation to nesting and brood-rearing habitats. J. Wildl. Manag. 79, 296–310 (2015).
17. Millan, C. H., Develey, P. F. & Verdade, L. M. Stand-level management practices increase occupancy by birds in exotic Eucalyptus plantations. For. Ecol. Manag. 336, 174–182 (2015).

18. Govindan, B. N. & Swihart, R. K. Community structure of acorn weevils (Curculio): Inferences from multispecies occupancy models. Can. J. Zool. 93, 31–39 (2014).

19. Higa, M. et al. Mapping large-scale bird distributions using occupancy models and citizen data with spatially biased sampling effort. Divers. Distrib. 21, 46–54 (2015).

20. Homyack, J. A., O'Bryan, C. J., Thornton, J. E. & Baldwin, R. F. Anuran assemblages associated with roadside ditches in a managed pine landscape. For. Ecol. Manag. 334, 217–231 (2014).

21. Muncy, B. L., Price, S. J., Bonner, S. J. & Barton, C. D. Mountaintop removal mining reduces stream salamander occupancy and richness in southeastern Kentucky (USA). Biol. Conserv. 180, 115–121 (2014).

22. Beesley, L. S. et al. Juvenile fish response to wetland inundation: How antecedent conditions can inform environmental flow policies for native fish. J. Appl. Ecol. 51, 1613–1621 (2014).

23. Mata, L., Goula, M. & Hahs, A. K. Conserving insect assemblages in urban landscapes: accounting for species-specific responses and imperfect detection. J. Insect Conserv. 18, 885–894 (2014).

24. Gilroy, J. J., Edwards, F. A., Medina Uribe, C. A., Haugaasen, T. & Edwards, D. P. Surrounding habitats mediate the trade-off between land-sharing and land-sparing agriculture in the tropics. J. Appl. Ecol. 51, 1337–1346 (2014).

25. Kroll, A. J. et al. Avian community composition associated with interactions between local and landscape habitat attributes. For. Ecol. Manag. 326, 46–57 (2014).

26. Rovero, F., Martin, E., Rosa, M., Ahumada, J. A. & Spitale, D. Estimating species richness and modelling habitat preferences of tropical forest mammals from camera trap data. PLoS ONE 9, (2014).

27. Pacifici, K., Zipkin, E. F., Collazo, J. A., Irizarry, J. I. & Dewan, A. Guidelines for a priori grouping of species in hierarchical community models. Ecol. Evol. 4, 877–888 (2014).

28. McManamay, R. A., Orth, D. J. & Jager, H. I. Accounting for variation in species detection in fish community monitoring. Fish. Manag. Ecol. 21, 96–112 (2014).

29. Prado, V. H. M. & Rossa-Feres, D. D. C. Multiple determinants of anuran richness and occurrence in an agricultural region in south-eastern Brazil. Environ. Manage. 53, 823–837 (2014).

30. Casanovas, P., Lynch, H. J. & Fagan, W. F. Using citizen science to estimate lichen diversity. Biol. Conserv. 171, 1–8 (2014).

31. Linden, D. W. & Roloff, G. J. Retained structures and bird communities in clearcut forests of the Pacific Northwest, USA. For. Ecol. Manag. 310, 1045–1056 (2013).

32. White, A. M., Zipkin, E. F., Manley, P. N. & Schlesinger, M. D. Simulating avian species and foraging group responses to fuel reduction treatments in coniferous forests. For. Ecol. Manag. 304, 261–274 (2013).

33. Guzy, J. C., Price, S. J. & Dorcas, M. E. The spatial configuration of greenspace affects semi-aquatic turtle occupancy and species richness in a suburban landscape. Landsc. Urban Plan. 117, 46–56 (2013).

34. Chandler, R. B. et al. A small-scale land-sparing approach to conserving biological diversity in tropical agricultural landscapes. Conserv. Biol. 27, 785–795 (2013).

35. Henden, J. A., Yoccoz, N. G., Ims, R. A. & Langeland, K. How spatial variation in areal extent and configuration of labile vegetation states affect the riparian bird community in Arctic tundra. PLoS ONE 8, (2013).

36. White, A. M., Zipkin, E. F., Manley, P. N. & Schlesinger, M. D. Conservation of avian diversity in the Sierra Nevada: Moving beyond a single-species management focus. PLoS ONE 8, (2013).

37. Holt, B. G., Rioja-Nieto, R., Aaron Macneil, M., Lupton, J. & Rahbek, C. Comparing diversity data collected using a protocol designed for volunteers with results from a professional alternative. Methods Ecol. Evol. 4, 383–392 (2013).

38. Giovanini, J., Kroll, A. J., Jones, J. E., Altman, B. & Arnett, E. B. Effects of management intervention on post-disturbance community composition: An experimental analysis using Bayesian Hierarchical Models. PLoS ONE 8, (2013).

39. Tingley, M. W. & Beissinger, S. R. Cryptic loss of montane avian richness and high community turnover over 100 years. Ecology 94, 598–609 (2013).

40. Chen, G., Kéry, M., Plattner, M., Ma, K. & Gardner, B. Imperfect detection is the rule rather than the exception in plant distribution studies. J. Ecol. 101, 183–191 (2013).

41. Hunt, S. D. et al. Responses of riparian reptile communities to damming and urbanization. Biol. Conserv. 157, 277–284 (2013).

42. Sauer, J. R., Blank, P. J., Zipkin, E. F., Fallon, J. E. & Fallon, F. W. Using multi-species occupancy models in structured decision making on managed lands. J. Wildl. Manag. 77, 117–127 (2013).

43. Zipkin, E. F., Campbell Grant, E. H. & Fagan, W. F. Evaluating the predictive abilities of community occupancy models using AUC while accounting for imperfect detection. Ecol. Appl. 22, 1962–1972 (2012).

44. Jones, J. E. et al. Avian species richness in relation to intensive forest management practices in early seral tree plantations. PLoS ONE 7, (2012).

45. Broms, K. M., Hooten, M. B. & Fitzpatrick, R. M. Accounting for imperfect detection in Hill numbers for biodiversity studies. Methods Ecol. Evol. 6, 99–108 (2015).

46. Ruiz-Gutiérrez, V. & Zipkin, E. F. Detection biases yield misleading patterns of species persistence and colonization in fragmented landscapes. Ecosphere 2, (2011).

47. Walls, S. C., Waddle, J. H. & Dorazio, R. M. Estimating occupancy dynamics in an anuran assemblage from Louisiana, USA. J. Wildl. Manag. 75, 876–882 (2011).

48. Yamaura, Y. et al. Modelling community dynamics based on species-level abundance models from detection/nondetection data. J. Appl. Ecol. 48, 67–75 (2011).

49. Wells, K., Böhm, S. M., Boch, S., Fischer, M. & Kalko, E. K. V. Local and landscape-scale forest attributes differ in their impact on bird assemblages across years in forest production land-scapes. Basic Appl. Ecol. 12, 97–106 (2011).

50. Holtrop, A. M., Cao, Y. & Dolan, C. R. Estimating sampling effort required for characterizing species richness and site-to-site similarity in fish assemblage surveys of Wadeable Illinois streams. Trans. Am. Fish. Soc. 139, 1421–1435 (2010).

51. Dorazio, R. M., Kéry, M., Royle, J. A. & Plattner, M. Models for inference in dynamic metacommunity systems. Ecology 91, 2466–2475 (2010).

52. Ruiz-Gutiérrez, V., Zipkin, E. F. & Dhondt, A. A. Occupancy dynamics in a tropical bird community: Unexpectedly high forest use by birds classified as non-forest species. J. Appl. Ecol. 47, 621–630 (2010).

53. Zipkin, E. F., Andrew Royle, J., Dawson, D. K. & Bates, S. Multi-species occurrence models to evaluate the effects of conservation and management actions. Biol. Conserv. 143, 479–484 (2010).

54. Zipkin, E. F., Dewan, A. & Andrew Royle, J. Impacts of forest fragmentation on species richness: A hierarchical approach to community modelling. J. Appl. Ecol. 46, 815–822 (2009).

55. Russell, R. E. et al. Modeling the effects of environmental disturbance on wildlife communities: Avian responses to prescribed fire. Ecol. Appl. 19, 1253–1263 (2009).

56. Homyack, J. A., O'Bryan, C. J., Thornton, J. E. & Baldwin, R. F. Community occupancy of herpetofauna in roadside ditches in a managed pine landscape. For. Ecol. Manag. 361, 346–357 (2016).

57. Kéry, M., Royle, J. A., Plattner, M. & Dorazio, R. M. Species richness and occupancy estimation in communities subject to temporary emigration. Ecology 90, 1279–1290 (2009).

58. Kery, M. & Royle, J. A. Inference about species richness and community structure using species-specific occupancy models in the national Swiss breeding bird survey MHB. 3, (2009).

59. Kéry, M. & Royle, J. A. Hierarchical Bayes estimation of species richness and occupancy in spatially replicated surveys. J. Appl. Ecol. 45, 589–598 (2008).

60. Dorazio, R. M., Royle, J. A., Söderström, B. & Glimskär, A. Estimating species richness and accumulation by modeling species occurrence and detectability. Ecology 87, 842–854 (2006).

61. Karenyi, N., Nel, R., Altwegg, R. & Sink, K. Incorporating species detectability into conservation targets based on the species-area relationship. Divers. Distrib. 22, 758–769 (2016).

62. Broms, K. M., Hooten, M. B. & Fitzpatrick, R. M. Model selection and assessment for multi-species occupancy models. Ecology 97, 1759–1770 (2016).

63. Laske, S. M. et al. Surface water connectivity drives richness and composition of Arctic lake fish assemblages. Freshw. Biol. 61, 1090–1104 (2016).

64. Beaudrot, L. et al. Limited carbon and biodiversity co-benefits for tropical forest mammals and birds. Ecol. Appl. 26, 1098–1111 (2016).

65. La, V. T. & Nudds, T. D. Estimation of avian species richness: Biases in morning surveys and efficient sampling from acoustic recordings. Ecosphere 7, (2016).

66. Dybala, K. E., Truan, M. L. & Engilis, A. Summer vs. Winter: Examining the temporal distribution of avian biodiversity to inform conservation. Condor 117, 560–576 (2015).

67. Eakin, C. J. et al. Avian response to green roofs in urban landscapes in the Midwestern USA. Wildl. Soc. Bull. 39, 574–582 (2015).

68. M'Gonigle, L. K., Ponisio, L. C., Cutler, K. & Kremen, C. Habitat restoration promotes pollinator persistence and colonization in intensively managed agriculture. Ecol. Appl. 25, 1557–1565 (2015).

69. Crosby, A. D., Elmore, R. D., Leslie, D. M. & Will, R. E. Looking beyond rare species as umbrella species: Northern Bobwhites (Colinus virginianus) and conservation of grassland and shrubland birds. Biol. Conserv. 186, 233–240 (2015).

70. Gilroy, J. J., Medina Uribe, C. A., Haugaasen, T. & Edwards, D. P. Effect of scale on trait predictors of species responses to agriculture. Conserv. Biol. 29, 463–472 (2015).

71. Walls, S. C. et al. Anuran site occupancy and species richness as tools for evaluating restoration of a hydrologically-modified landscape. Wetl. Ecol. Manag. 22, 625–639 (2014).

72. Sanderlin, J. S., Block, W. M. & Ganey, J. L. Optimizing study design for multi-species avian monitoring programmes. J. Appl. Ecol. 51, 860–870 (2014).

73. Walls, S. C., Waddle, J. H. & Faulkner, S. P. Wetland reserve program enhances site occupancy and species richness in assemblages of anuran amphibians in the Mississippi Alluvial Valley, USA. Wetlands 34, 197–207 (2014).

74. Grant, E. H. C., Zipkin, E. F., Nichols, J. D. & Campbell, J. P. A strategy for monitoring and managing declines in an amphibian community. Conserv. Biol. 27, 1245–1253 (2013).

75. Zhao, Q., Azeria, E. T., Le Blanc, M. L., Lemaître, J. & Fortin, D. Landscape-scale disturbances modified bird community. PLoS ONE 8, (2013).

76. Kalies, E. L. & Rosenstock, S. S. Stand structure and breeding birds: Implications for restor-ing ponderosa pine forests. J. Wildl. Manag. 77, 1157–1165 (2013).

77. Mattsson, B. J. et al. Explaining local-scale species distributions: Relative contributions of spatial autocorrelation and landscape heterogeneity for an avian assemblage. PLoS ONE 8, (2013).

78. Cheal, A. J., Emslie, M., MacNeil, M. A., Miller, I. & Sweatman, H. Spatial variation in the functional characteristics of herbivorous fish communities and the resilience of coral reefs. Ecol. Appl. 23, 174–188 (2013).

79. Linden, D. W., Roloff, G. J. & Kroll, A. J. Conserving avian richness through structure retention in managed forests of the Pacific Northwest, USA. For. Ecol. Manag. 284, 174–184 (2012).

80. Yamaura, Y. et al. Biodiversity of man-made open habitats in an underused country: A class of multispecies abundance models for count data. Biodivers. Conserv. 21, 1365–1380 (2012).

81. Yue, S., Brodie, J. F., Zipkin, E. F. & Bernard, H. Oil palm plantations fail to support mammal diversity. Ecol. Appl. 25, 2285–2292 (2015).

82. Waddle, J. H., Glorioso, B. M. & Faulkner, S. P. A quantitative assessment of the conservation benefits of the wetlands reserve program to amphibians. Restor. Ecol. 21, 200–206 (2013).

83. Brodie, J. F. et al. Correlation and persistence of hunting and logging impacts on tropical rainforest mammals. Conserv. Biol. 29, 110–121 (2015).

84. Sanderlin, J. S., Block, W. M., Ganey, J. L. & Iniguez, J. M. Preliminary assessment of species richness and avian community dynamics in the Madrean Sky Islands, Arizona. USDA Foreset Serv. Proc. 180–190 (2013).

85. Yamaura, Y. et al. Estimating species – area relationships by modeling abundance and frequency subject to incomplete sampling. Ecol. Evol. 6, 4836–4848 (2016).

86. Pandolfo, T. J. et al. Species traits and catchment-scale habitat factors influence the occurrence of freshwater mussel populations and assemblages. Freshw. Biol. 61, 1671–1684 (2016).

87. Potoka, K. M., Shea, C. P. & Bettoli, P. W. Multispecies occupancy modeling as a tool for evaluating the status and distribution of darters in the Elk River, Tennessee. Trans. Am. Fish. Soc. 145, 1110–1121 (2016).

88. Sanderlin, J. S., Block, W. M. & Strohmeyer, B. E. Long-term post-wildfire correlates with avian community dynamics in ponderosa pine forests [Chapter J]. in (ed. Ralston, B. E.) (U.S. Geological Survey Scientific Investigations Report 2015-5180, 2016).

89. Riffell, S. K. et al. Response of non-grassland avian guilds to adjacent herbaceous field buffers: Testing the configuration of targeted conservation practices in agricultural landscapes. J. Appl. Ecol. 52, 300–309 (2015).
