# Supplementary material for: Inferring species richness using multispecies occupancy modeling: Estimation performance and interpretation
Source: Ecol Evol. 2019 Feb 5;9(2):780–92. doi: 10.1002/ece3.4821 (PMC6362448; doi:10.1002/ece3.4821)

SIMULATION REPORT

Simulation settings

seed = 50, nspecies = 100
S = 150, K = 2, nz = 50 (number of all-zero species, for data augmentation)
psi = scenario psiH (betas: mu = -1, sd = 0.3)
p = scenario 1 (alphas: mu = -2, sd = 1)
prior settings: 3
mcmc settings: ni = 225000, nt = 25, nb = 175000, nc = 3

Time and convergence

This simulation took 204 minutes to run.
THIS SIMULATION HAS CONVERGED (looking at mu.lpsi, mu.lp, sd.lpsi, sd.lp, and omega).
There are Rhat values up to 3.64. The Rhat for Ntotal is 3.64.

Missed species

A total of 2 species were missed in the sampling. The expectation was to miss about 3.1 species.

Simulated and estimated occupancy and detectabilities

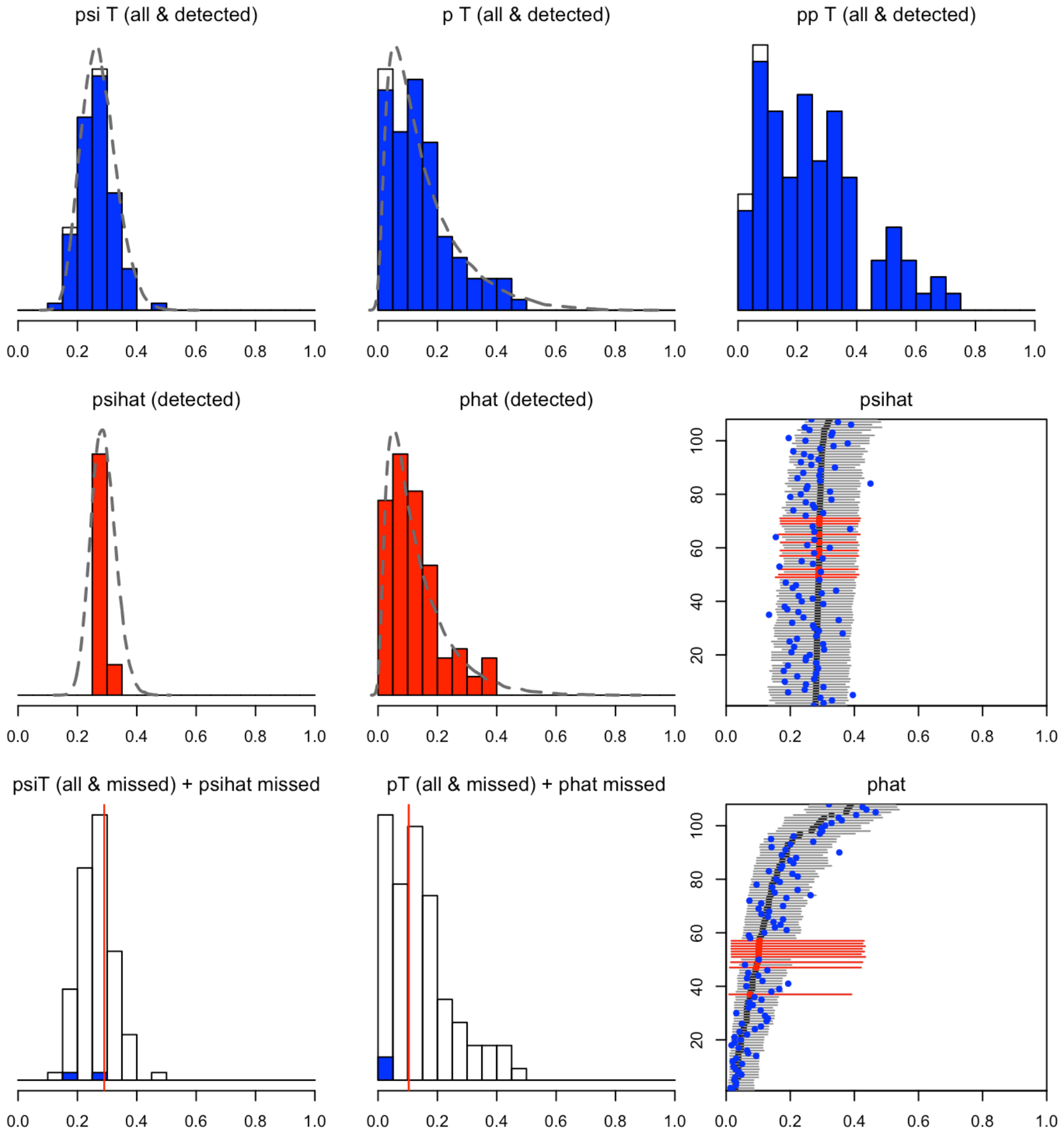

Convergence checks

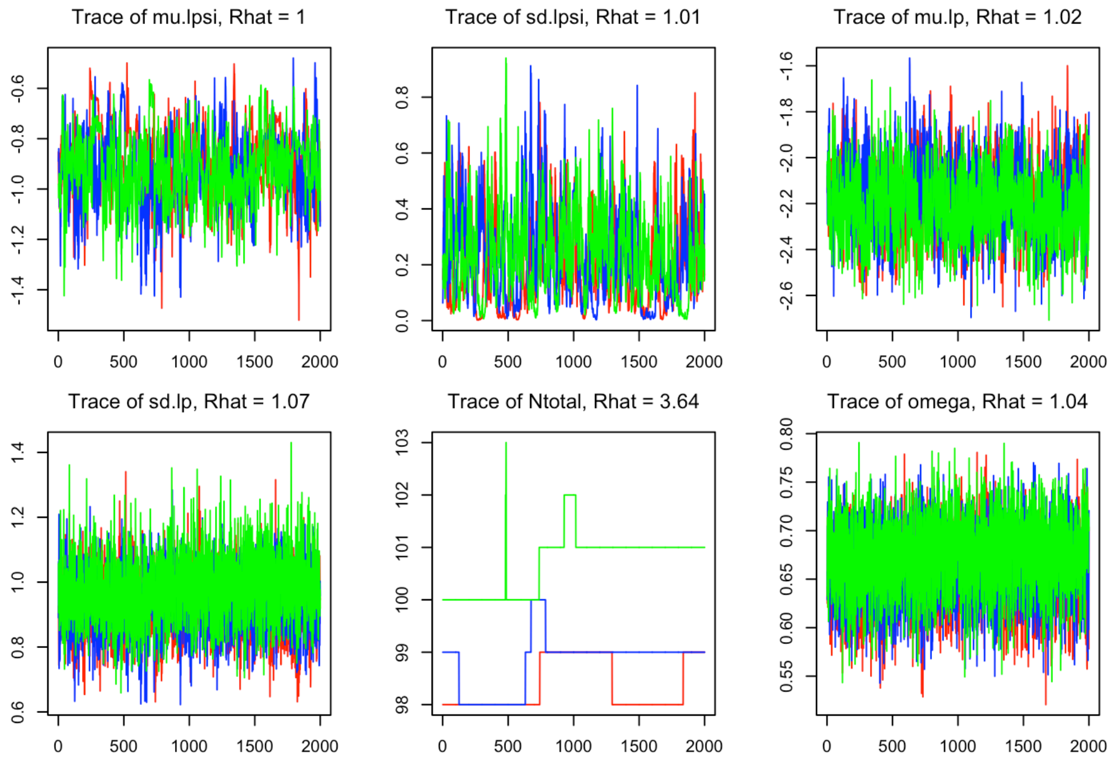

Estimated number of species

Nhat = 99 (98,101)
Nhat_small = 99 (98,101)

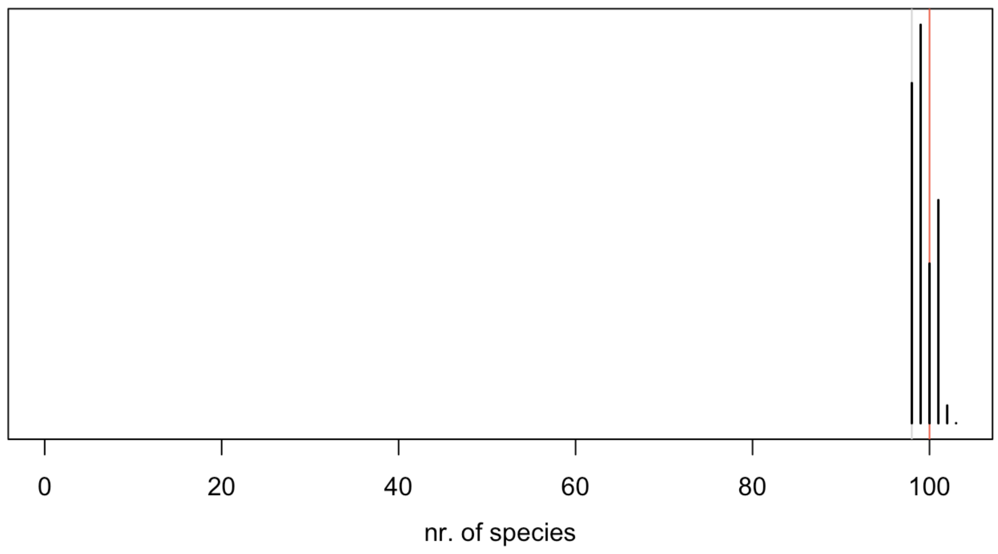

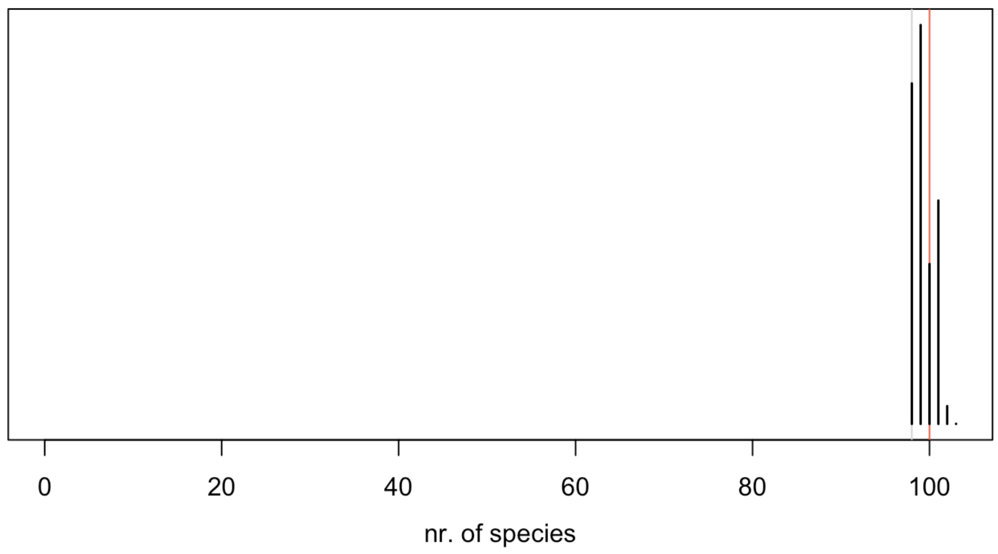

Supplement: Supplementary file 3 [file ECE3-9-780-s003.pdf]
