# Supplementary material for: Inferring species richness using multispecies occupancy modeling: Estimation performance and interpretation
Source: Ecol Evol. 2019 Feb 5;9(2):780–92. doi: 10.1002/ece3.4821 (PMC6362448; doi:10.1002/ece3.4821)
Supplement: Supplementary file 4 [file ECE3-9-780-s004.pdf]

Appendix S4 – Distributions used to generate species detection probabilities in simulated data

Case ID	Key features	Type	Details	Pdf
'Det1'	Reference	Logit-normal	$p_k = 1/(1+e^{-x})$, where: $x \sim \mathrm{N}(-2,1)$	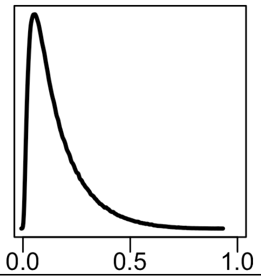
'Det2'	Steep decays	Scaled beta	$p_k = 0.3x + 0.02$, where: $x \sim \mathrm{Beta}(1.05,1.05)$	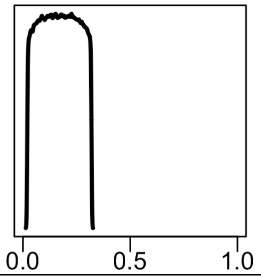
'Det3'	Bimodal, steep decays	Mixture of scaled betas	$p_k = \pi y_1 + (1-\pi) y_2$, where: $y_1 = 0.15x_1 + 0.1,$ $y_2 = 0.3x_2 + 0.6,$ $x_1 \sim \mathrm{Beta}(2,2)$ $x_2 \sim \mathrm{Beta}(2,2)$ $\pi \sim \mathrm{Bernoulli}(0.8)$	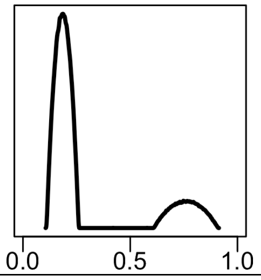
'Det4'	Bimodal	Mixture of logit-normals	$p_k = \pi y_1 + (1-\pi) y_2$, where: $y_1 = 1/(1+e^{-x_1}),$ $y_2 = 1/(1+e^{-x_2}),$ $x_1 \sim \mathrm{N}(-2.5,0.8)$ $x_2 \sim \mathrm{N}(0,0.2)$ $\pi \sim \mathrm{Bernoulli}(0.5)$	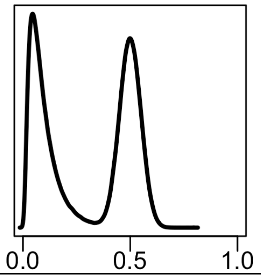
'Det5'	Unimodal, fat tails	Truncated t distribution	$p_k = \begin{cases} 0.1x + 0.5, & \text{if } x \in [-4.8, 5.0] \\ 0.02, & \text{if } x < -4.8 \\ 1, & \text{if } x > 5.0 \end{cases}$ where $x \sim t(1)$	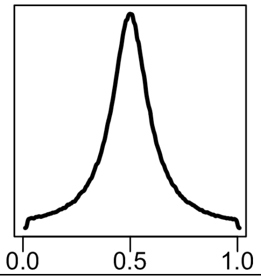
'Det6'	Steep decays	Scaled beta	$p_k = 0.6x + 0.2$, where: $x \sim \mathrm{Beta}(1.1,1.1)$	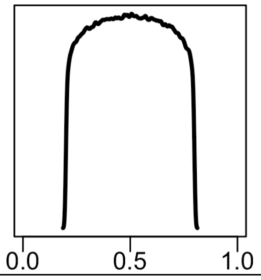
