# Supplementary material for: Inferring species richness using multispecies occupancy modeling: Estimation performance and interpretation
Source: Ecol Evol. 2019 Feb 5;9(2):780–92. doi: 10.1002/ece3.4821 (PMC6362448; doi:10.1002/ece3.4821)
Supplement: Supplementary file 5 [file ECE3-9-780-s005.docx]

***** RichnessEval_functs_analysis.R *****

###########################################################################################

###########################################################################################

#### MSODM richness estimation evaluation analyses

#### --------------------------------------------------------------------------------------

#### FUNCTIONS FOR DATA GENERATION (simulated data) AND ANALYSES

#### (version 18/Feb/2018)

###########################################################################################

###########################################################################################

###########################################################################################

## Generate simple simulated PA data for a community of species (no predictors)

###########################################################################################

genSimpleLandscape<-function(nsites=1e4,nspecies=100,betas_mu=-1,betas_sd=0.3){

betas<-rnorm(n=nspecies, betas_mu, betas_sd)

psi<- rep(1,nsites) %o% plogis(betas) # occupancy probabilities

tmp<-matrix(runif(nspecies*nsites),nrow=nsites,ncol=nspecies)

Z<-(tmp<psi)*1 # species PA

mylandscape<-data.frame(Z=Z,psi=psi) # bundle info in a dataframe

return(mylandscape)

}

###########################################################################################

## Generate simulated detection/non-detection data for a given community of species

## Constant detectability - different parametric forms to define variation in p across sp.

## If "ideal" (=normal, "case_p=1"), can select mean and sd (alphas_mu or alphas_sd)

## Other cases disregard these params, and have fixed parameters (details in paper)

###########################################################################################

genSampling<-function(mylandscape,S=90,K=2,alphas_mu=-2,alphas_sd=0.4,case_p=1){

# get info about dimensions

nsites<-nrow(mylandscape)

nspecies<-length(grep("Z.", colnames(mylandscape)))

# number of visits per site (here for all sites the same number, K)

nrep<-rep(K,S)

# select the sites to sample

id<-sample(1:nsites,size=S,replace=F)

mydata<-mylandscape[id,]

# simulate detection probabilities

if (case_p==1){ #NORMAL IN LOGIT SCALE ("IDEAL")

alphas<-rnorm(n=nspecies, alphas_mu, alphas_sd)

p<-plogis(alphas)

}

if (case_p==2){ #STEEP DECAY

p<-rbeta(n=nspecies,shape1=1.05,shape2=1.05)*0.3+0.02

}

if (case_p==3){ #BIMODAL, STEEP DECAY (one small bump high)

tmp<-c(((rbeta(n=4*nspecies,shape1=2,shape2=2))*0.15+0.1),(rbeta(n=nspecies,shape1=2,shape2=2))*0.3+0.6)+0.01

tmp<-tmp[(tmp>0.02)&(tmp<1)]

p<-sample(tmp,size=nspecies,replace=T)

}

if (case_p==4){ #BIMODAL, MIXTURE OF NORMALS

tmp1<-1/(1+exp(-rnorm(n=nspecies,-2.5,0.8)));

tmp2<-1/(1+exp(-rnorm(n=nspecies,0,0.2)));

p<-sample(c(tmp1,tmp2),size=nspecies,replace=T)

}

if (case_p==5){ #UNIMODAL, FATTER TAILS

tmp<-rt(n=nspecies, df=1)/10+0.5

tmp<-tmp[(tmp>0.02)&(tmp<1)]

p<-sample(tmp,size=nspecies,replace=T)

}

if (case_p==6){ #FLAT: ~U(0.2-0.8) w round corners

p<-rbeta(n=nspecies,shape1=1.1,shape2=1.1)*0.6+0.2

}

# simulate species detections

p<-matrix(p,nrow=S,ncol=length(p),byrow=T)

pu<-p*mydata[,grep("Z.", colnames(mydata))]

y<-matrix(NA,nrow=S,ncol=nspecies)

for (ii in 1:S){

for (jj in 1:nspecies){

y[ii,jj]<-rbinom(n=1,size=nrep[ii],pu[ii,jj])

}

}

# bundle info in a dataframe

mydata<-cbind(mylandscape[id,],y=y,p=p,nrep=nrep)

}

###########################################################################################

## Analyze community det/non-det data in JAGS (model without predictors)

###########################################################################################

MSODM_constant<-function(nz=100,mydata,removeall0=T,nt=4,nb=1e4,nc=3,params=c("Ntotal"),

modelname="MSODM_JAGS.txt",ni_max=1e5,nincr=5e4){

# extract survey data

yALL<-y<-mydata[,grep("y.", colnames(mydata))]

if (removeall0){ IDout<-which(colSums(yALL)==0)

}else{IDout<-c()}

if (length(IDout>0)) y<-as.matrix(yALL[,-IDout]) #remove species not recorded in dataset

# get info about dimensions

n<-ncol(y)

S<-nrow(y)

# augment data set

M <- n + nz # size of augmented data set ('superpopulation')

yaug <- cbind(y, array(0, dim=c(S, nz)))

# bundle data set

sim.data <- list(yaug=yaug, nsite=S, nrep=mydata$nrep, M=M, n=n, nz=nz)

# initial values

wst <- c(rep(1, n),rep(0, nz)) #as if no species missed

zst <- (yaug>0)*1 #init z to det/non-det observations

inits <- function() list(z=zst, w=wst, lpsi=rnorm(n=n+nz), lp=rnorm(n=n+nz),

mu.lpsi=rnorm(1), mu.lp=rnorm(1), sd.lpsi=runif(n=1,min=0.5,max=2), sd.lp=runif(n=1,min=0.5,max=2))

# call JAGS from R

model.out <- autojags(sim.data, inits, params, modelname, n.chains=nc, n.burnin=nb, n.thin=nt, max.iter=(nb+ni_max),

iter.increment=nincr, save.all.iter=F, store.data=F, codaOnly=F, Rhat.limit=1.1, verbose=T, parallel=T)

return(list(model.out=model.out,IDout=IDout))

}

###########################################################################################

## Specify model in JAGS language (based on AHM_model9, from Kery & Royle book)

###########################################################################################

sink("MSODM_JAGS.txt")

cat("

model {

# Priors to describe heterogeneity among species in community

for (k in 1:M){ # Loop over all species in augmented list

lpsi[k] ~ dnorm(mu.lpsi, tau.lpsi)

lp[k] ~ dnorm(mu.lp, tau.lp)

}

# Hyperpriors to describe full community

omega ~ dunif(0,1) # For data augmentation (probability of community membership)

mu.lpsi ~ dnorm(0,0.001) # Community mean of occupancy (logit)

mu.lp ~ dnorm(0,0.001) # Community mean of detection (logit)

tau.lpsi <- pow(sd.lpsi, -2)

sd.lpsi ~ dunif(0,5) # Species heterogeneity in logit(psi)

tau.lp <- pow(sd.lp, -2)

sd.lp ~ dunif(0,5) # Species heterogeneity in logit(p)

# Superpopulation process

for (k in 1:M){

w[k] ~ dbern(omega) # Community membership indicator

}

# Ecological model for latent occurrence z (process model)

for (k in 1:M){

mu.psi[k] <- w[k] * psi[k] # species not part of community zeroed out for z

logit(psi[k]) <- lpsi[k]

for (i in 1:nsite) {

z[i,k] ~ dbern(mu.psi[k])

}

}

# Observation model for observed detection frequencies

for (k in 1:M){

logit(p[k]) <- lp[k]

for (i in 1:nsite) {

mu.p[i,k] <- z[i,k] * p[k] # non-occurring species are zeroed out for p

yaug[i,k] ~ dbin(mu.p[i,k], nrep[i])

}

}

# Derived quantities

for (k in 1:M){

Socc.fs[k] <- sum(z[,k]) # Number of occupied sites among the sampled ones

speciesP[k]<-ifelse(Socc.fs[k]>0,1,0)

}

Nsmall<-sum(speciesP)

for (i in 1:nsite) {

Nsite[i] <- sum(z[i,]) # Number of occurring species at each site

}

n0 <- sum(w[(n+1):(n+nz)]) # Number of undetected species

Ntotal <- sum(w[]) # Total metacommunity size (= nspec + n0)

# Some other things to monitor (subset to control file size)

w10missed<-w[(n+1):(n+10)]

psi10missed<-psi[(n+1):(n+10)]

p10missed<-p[(n+1):(n+10)]

psi.D<-psi[1:n]

p.D<-p[1:n]

}

",fill = TRUE)

sink()

###########################################################################################

## Same as above but with different (narrower) priors for regression coefficients

###########################################################################################

sink("MSODM_JAGS_v2.txt")

cat("

model {

# Priors to describe heterogeneity among species in community

for (k in 1:M){ # Loop over all species in augmented list

lpsi[k] ~ dnorm(mu.lpsi, tau.lpsi)

lp[k] ~ dnorm(mu.lp, tau.lp)

}

# Hyperpriors to describe full community

omega ~ dunif(0,1) # For data augmentation (probability of community membership)

mu.lpsi ~ dnorm(0,0.2) # Community mean of occupancy (logit)

mu.lp ~ dnorm(0,0.2) # Community mean of detection (logit)

tau.lpsi <- pow(sd.lpsi, -2)

sd.lpsi ~ dunif(0,5) # Species heterogeneity in logit(psi)

tau.lp <- pow(sd.lp, -2)

sd.lp ~ dunif(0,5) # Species heterogeneity in logit(p)

# Superpopulation process

for (k in 1:M){

w[k] ~ dbern(omega) # Community membership indicator

}

# Ecological model for latent occurrence z (process model)

for (k in 1:M){

mu.psi[k] <- w[k] * psi[k] # species not part of community zeroed out for z

logit(psi[k]) <- lpsi[k]

for (i in 1:nsite) {

z[i,k] ~ dbern(mu.psi[k])

}

}

# Observation model for observed detection frequencies

for (k in 1:M){

logit(p[k]) <- lp[k]

for (i in 1:nsite) {

mu.p[i,k] <- z[i,k] * p[k] # non-occurring species are zeroed out for p

yaug[i,k] ~ dbin(mu.p[i,k], nrep[i])

}

}

# Derived quantities

for (k in 1:M){

Socc.fs[k] <- sum(z[,k]) # Number of occupied sites among the sampled ones

speciesP[k]<-ifelse(Socc.fs[k]>0,1,0)

}

Nsmall<-sum(speciesP)

for (i in 1:nsite) {

Nsite[i] <- sum(z[i,]) # Number of occurring species at each site

}

n0 <- sum(w[(n+1):(n+nz)]) # Number of undetected species

Ntotal <- sum(w[]) # Total metacommunity size (= nspec + n0)

# Some other things to monitor (subset to control file size)

w10missed<-w[(n+1):(n+10)]

psi10missed<-psi[(n+1):(n+10)]

p10missed<-p[(n+1):(n+10)]

psi.D<-psi[1:n]

p.D<-p[1:n]

}

",fill = TRUE)

sink()

###########################################################################################

## Same as above but with different (narrower) priors for regression coefficients and

## also for parameter Omega (following Link 2013)

###########################################################################################

sink("MSODM_JAGS_v3.txt")

cat("

model {

# Priors to describe heterogeneity among species in community

for (k in 1:M){ # Loop over all species in augmented list

lpsi[k] ~ dnorm(mu.lpsi, tau.lpsi)

lp[k] ~ dnorm(mu.lp, tau.lp)

}

# Hyperpriors to describe full community

omega ~ dbeta(0.001,1) # For data augmentation (probability of community membership)

mu.lpsi ~ dnorm(0,0.2) # Community mean of occupancy (logit)

mu.lp ~ dnorm(0,0.2) # Community mean of detection (logit)

tau.lpsi <- pow(sd.lpsi, -2)

sd.lpsi ~ dunif(0,5) # Species heterogeneity in logit(psi)

tau.lp <- pow(sd.lp, -2)

sd.lp ~ dunif(0,5) # Species heterogeneity in logit(p)

# Superpopulation process

for (k in 1:M){

w[k] ~ dbern(omega) # Community membership indicator

}

# Ecological model for latent occurrence z (process model)

for (k in 1:M){

mu.psi[k] <- w[k] * psi[k] # species not part of community zeroed out for z

logit(psi[k]) <- lpsi[k]

for (i in 1:nsite) {

z[i,k] ~ dbern(mu.psi[k])

}

}

# Observation model for observed detection frequencies

for (k in 1:M){

logit(p[k]) <- lp[k]

for (i in 1:nsite) {

mu.p[i,k] <- z[i,k] * p[k] # non-occurring species are zeroed out for p

yaug[i,k] ~ dbin(mu.p[i,k], nrep[i])

}

}

# Derived quantities

for (k in 1:M){

Socc.fs[k] <- sum(z[,k]) # Number of occupied sites among the sampled ones

speciesP[k]<-ifelse(Socc.fs[k]>0,1,0)

}

Nsmall<-sum(speciesP)

for (i in 1:nsite) {

Nsite[i] <- sum(z[i,]) # Number of occurring species at each site

}

n0 <- sum(w[(n+1):(n+nz)]) # Number of undetected species

Ntotal <- sum(w[]) # Total metacommunity size (= nspec + n0)

# Some other things to monitor (subset to control file size)

w10missed<-w[(n+1):(n+10)]

psi10missed<-psi[(n+1):(n+10)]

p10missed<-p[(n+1):(n+10)]

psi.D<-psi[1:n]

p.D<-p[1:n]

}

",fill = TRUE)

sink()

***** RichnessEval_main.R *****

###########################################################################################

###########################################################################################

#### MSODM richness estimation evaluation analyses

#### --------------------------------------------------------------------------------------

#### MAIN SCRIPT TO RUN ONE ANALYSES (simulation of one scenario)

#### (version 18/Feb/2018)

###########################################################################################

###########################################################################################

t1<-proc.time()

set.seed(myseed)

# simulate data

mylandscape<-genSimpleLandscape(nspecies=nspecies,betas_mu=betas_mu,betas_sd=betas_sd)

mydata<-genSampling(mylandscape,S=S,K=K,alphas_mu=alphas_mu,alphas_sd=alphas_sd,case_p=case_p)

#analyse data

myres<-MSODM_constant(nz=nz,mydata,removeall0=T,params=params,ni=ni,nt=nt,nb=nb,nc=3,

modelname=mymodelname,ni_max=ni_max,nincr=nincr)

model.out<-myres$model.out; IDout<-myres$IDout

rm(myres)

t2<-(proc.time()-t1)[3]; print(paste0("this sim took ",round(t2/60)," min"))

#save results

fname<-paste0("N",nspecies,"_p",case_p,"_",case_psi,"_S",S,"_J",K,"_m",case_prior,"_d",sprintf("%02d", seedii))

resfname<-paste0("00_sims/",fname,"_",sprintf("%d", ni_max),"_",nz,".RData")

save.image(file=resfname)

***** RichnessEval_throw.R *****

###########################################################################################

###########################################################################################

#### MSODM richness estimation evaluation analyses

#### --------------------------------------------------------------------------------------

#### EXAMPLE OF FILE TO RUN A SET OF SIMULATIONS

#### (version 18/Feb/2018)

###########################################################################################

###########################################################################################

##clean space and load functions #########################################################

rm(list=ls())

library(jagsUI)

source("RichnessEval_functs_analysis.R") #load functions

###########################################################################################

####### CHOSE HERE THE SIMULATION PARAMETERS ##############################################

nspecies=100 #true number of species

case_psi="psiH" #occupancy scenario

case_p=1 #detectability scenario

case_prior=1 #which version of priors to use

ni_max=2e5 #max nr of MCMC samples

seediis=1:10 #which dataset/s to generate (choose a seed in our set)

params_type=3

save_data=T

###########################################################################################

###########################################################################################

myseeds_v<-c(1010,10,50,2342,535,99,987,1142,888,1209)

# set up community parameters

if (case_p==1){alphas_mu=-2;alphas_sd=1} #detectability scenario

if (case_psi=="psiH"){ #occupancy scenario (HIGH)

betas_mu=-1;betas_sd=0.3;

nz_v<-c(500,100,100,100,50,50,50,50,50) #nzeros (based on preliminary analyses)

}

if (case_psi=="psiL"){ #occupancy scenario (LOW)

betas_mu=-2;betas_sd=0.6

nz_v<-c(500,500,500,500,200,200,100,50,50)

}

# set up MCMC

nincr=50e3 #sample increments

nb=25e3

nt=25

# set up model (different options for priors)

if (case_prior==1) {mymodelname<-"MSODM_JAGS.txt"}

if (case_prior==2) {mymodelname<-"MSODM_JAGS_v2.txt"} #narrower regression priors

if (case_prior==3) {mymodelname<-"MSODM_JAGS_v3.txt"} #narrower regression priors, a scale prior for omega

# params to store

params <- c("mu.lpsi","sd.lpsi","mu.lp","sd.lp","Ntotal","omega","Nsmall")

if (params_type>1) params<-c(params,"w10missed","psi10missed","p10missed")

if (params_type>2) params<-c(params,"psi.D","p.D")

# run the analyses (all combinations of S and K, for all seeds selected)

for (ii in 1:length(seediis)){

seedii<-seediis[ii];myseed=myseeds_v[seedii];

S=25;K=2;nz=nz_v[1];source("RichnessEval_main_v180218.R");

S=25;K=4;nz=nz_v[2];source("RichnessEval_main_v180218.R");

S=25;K=6;nz=nz_v[3];source("RichnessEval_main_v180218.R");

S=50;K=2;nz=nz_v[4];source("RichnessEval_main_v180218.R");

S=50;K=4;nz=nz_v[5]; source("RichnessEval_main_v180218.R");

S=50;K=6;nz=nz_v[6]; source("RichnessEval_main_v180218.R");

S=150;K=2;nz=nz_v[7];source("RichnessEval_main_v180218.R");

S=150;K=4;nz=nz_v[8];source("RichnessEval_main_v180218.R");

S=150;K=6;nz=nz_v[9];source("RichnessEval_main_v180218.R");

}
